# Associations of rumen and rectum bacteria with the sustained productive performance of dairy cows

**DOI:** 10.3389/fmicb.2025.1565034

**Published:** 2025-04-29

**Authors:** Jianhao Yang, Yifan Li, Mengkun Sun, Yuan Zhang, Shanshan Guo, Dong Zhou, Pengfei Lin, Aihua Wang, Yaping Jin

**Affiliations:** ^1^College of Veterinary Medicine, Northwest A&F University, Xianyang, China; ^2^Key Laboratory of Animal Biotechnology, Ministry of Agriculture and Rural Affairs, Northwest A&F University, Xianyang, China

**Keywords:** rumen bacteria, rectum bacteria, production performances, milk yield, productive lifespan

## Abstract

The gut bacterial community is essential for maintaining lifelong health and productivity in ruminants, but the relationship between the gut microbiota and the sustained productivity of ruminants remains inadequately understood. In this study, we selected long-lived dairy cows in mid-lactation (≥5 parities) with different levels of milk production (*n* = 10). Significant differences were observed in the rumen bacterial structures between the two groups of dairy cows, whereas no significant differences were detected in the rectum bacterial communities. Additionally, there were no significant differences in serum oxidative stress biomarkers, inflammatory markers, or immunological markers between the long-lived high-yield (LH) and long-lived low-yield (LL) dairy cows. Furthermore, the concentrations of propionate (Pr) in the rumen and butyrate (Bu) in the rectum were elevated in the high-yield group. Spearman correlation and microbial co-occurrence network analyses revealed that several rumen-enriched bacteria, such as *Syntrophococcus*, *Lachnospira*, *Shuttleworthia*, Erysipelotrichaceae_UCG-2, and *Roseburiaare* associated with rumen propionate (Pr) production. In the rectum, the reduced abundance of Christensenellaceae_R-7_group and *Moryella* favors butyrate production. Furthermore, Random Forest machine learning analysis demonstrated that six bacterial taxa in the rumen combined with one serum biomarker, as well as three bacterial taxa in the rectum combined with three serum biomarkers, can serve as potential biomarkers for distinguishing between LH and long- LL dairy cows, achieving prediction accuracies of 92 and 99%, respectively. The findings of this study indicate that rumen and rectum bacteria are associated with the milk production phenotypes of dairy cows with sustained productivity. The rumen microbes are closely linked to the long-term productive capacity of dairy cows and represent a key target for the development of gut microbiota-based interventions. The unique bacterial communities of the rumen and rectum of long-lived high-yielding dairy cows contribute to maintaining their productive capacity.

## 1 Introduction

Milk and dairy products are high-quality nutritional sources, providing energy, essential amino acids, fats, vitamins, and minerals, of the greatest benefit to people in developing countries in the fight against hunger and malnutrition (Headey, 2023). In 2023, the global dairy market was valued at $944.7 billion, and global dairy production (with milk accounting for approximately 81%) was projected to grow by 1.5% annually over the next decade. Therefore, improving dairy cow productivity and milk yield are key objectives for the future development of the global dairy industry (Organization for Economic Co-Operation and Development, Food and Agriculture Organization of the United Nations, 2023). The overall profitability of a dairy herd depends on the lifetime productivity of the cows, which is a trait that combines production level and longevity (Zhang H. et al., 2024). The profit per kilogram of fat and protein-corrected milk (FPCM) is positively correlated with productive lifespan. Extending the productive lifespan of dairy cows from 1 to 7 years reduces replacement costs from 38% to 9%, while increasing the lifetime production of milk, beef, and edible protein (Grandl et al., 2019). Additionally, extending the productive lifespan of dairy cows can decrease the need for replacement cows, thereby reducing associated feeding costs and greenhouse gas emissions, contributing to more sustainable milk production (Han et al., 2024). The average productive lifespan of dairy cows is typically 3 to 4 years, whereas their natural lifespan can reach approximately 20 years (De Vries, 2020; De Vries and Marcondes, 2020; Hu et al., 2021; Shrestha et al., 2025). The productive performance of older dairy cows declines with age, and high-yielding older cows are more susceptible to health issues, such as reproductive disorders, mastitis, and metabolic diseases, increasing the risk of involuntary culling, and reducing their productive lifespan and lifetime productivity (Pinedo et al., 2010; De Vries and Marcondes, 2020).

The rumen is the primary site for digestion and nutrient absorption in ruminants and the resident microbes are essential for optimal nutrition. Rumen microbial fermentation produces short-chain fatty acids (SCFAs), primarily acetate, propionate, and butyrate, which supply 70% of the daily energy requirements for ruminants (Yeoman and White, 2014). Acetate produced during rumen microbial fermentation participates in *de novo* fatty acid synthesis, along with medium-chain and long-chain fatty acids generated during feed degradation, jointly contributing to milk fat synthesis (Toral et al., 2018). Rumen microbes degrade fiber and starch in the feed to produce propionate, which serves as a precursor for glucose synthesis through hepatic gluconeogenesis, and the glucose can be subsequently used to produce lactose (Aschenbach et al., 2010). As the primary sugar component in milk, lactose concentration plays a crucial role in maintaining osmotic pressure within the mammary gland, driving the transfer of water from the bloodstream into milk, and sustaining milk production (Antanaitis et al., 2024). Butyrate produced during rumen fermentation serves as an important energy substrate for the rumen epithelial cells of dairy cows, stimulating their proliferation and reducing apoptosis. Additionally, butyrate supplementation enhances fatty acid synthesis and increases the concentrations of milk fat and milk protein (Xu et al., 2018; Kang et al., 2023; Francis et al., 2024). In addition, rumen microbes are a key source of high-quality protein for absorption in the small intestine and for milk protein synthesis (Larson, 1965). Studies on the differences in rumen microbial composition and functionality related to milk protein yield (MPY) have found that the microbial composition and activity of the rumen microbiota account for 18 and 22% of the variation in MPY, respectively (Xue et al., 2020). The relative abundances of members of the phyla Firmicutes and Bacteroidetes are significantly positively correlated with milk fat content (Jami et al., 2014; Si et al., 2023). Dysbiosis of the rumen microbiota promotes the development of mastitis induced by subclinical ketosis and subacute ruminal acidosis by allowing more LPS to enter the bloodstream and disrupting the blood-milk barrier (Hu et al., 2022; Zhao C. et al., 2023; Tang et al., 2024). In transition and ketotic cows, reduced populations of rumen bacteria such as *Succinivibrio ruminantium* and *Megasphaera elsdenii* may promote energy imbalance and the progression of ketosis (Wang et al., 2012).

In mammals, the hindgut microbiota plays a pivotal role in nutrient absorption, metabolism, and immune function. An increase in the abundance of *Bifidobacteria* in feces is associated with improvements in several dairy product characteristics, including milk yield, milk fat and protein yields (Brulin et al., 2024). Based on the relative abundance of microbial genera obtained by 16S rRNA gene sequencing, microbiability calculations can account for 43.1% of the variation in the postpartum serum oxidative stress index (Gu et al., 2023a). Alterations in the hindgut microbiota and their functions, particularly those related to secondary bile acid synthesis, inhibit monocyte function during excessive fat mobilization in transition cows, leading to immune suppression in the postpartum period (Gu et al., 2023b). Oxidative stress during this phase, coupled with immune suppression, increases the proportion of dairy cows with displaced abomasum and clinical ketosis by 3.6% (Ospina et al., 2010). Transplantation of fecal microbiota from cows with mastitis to mice has provided evidence that dysbiosis of the hindgut microbiota contributes to the development of mastitis and alters the mammary microbiota (Ma et al., 2018; Zhao et al., 2022). The rumen and hindgut microbiota are directly associated with dairy production traits, mammary health, and reproductive performance, significantly affecting the productive lifespan of cows. In a study of 2054 herds in the eastern United States, low production and mastitis each accounted for 12% of the reasons for cow culling, while reproductive disorders accounted for 18% (De Vries and Marcondes, 2020).

In this study, we hypothesized that cows with identical DMI and dietary compositions exhibit different metabolic phenotypes driven by the predominant bacteria of the rumen and rectum, thereby influencing their sustained productivity. To test this hypothesis, we selected high-yielding and low-yielding cows with extended productive lifespans. We determined the bacterial compositions of the rumen and rectum, predicted their functions, and measured rumen and rectum fermentation parameters, serum biomarkers, and milk production phenotypes for each group. The aim of the study was to identify the unique differences in the rumen and rectum microbiota of cows with long-term productive capacity and their potential impact on milk production phenotypes, and to provide new insights for selecting and breeding dairy cows to optimize the productive lifespan of dairy herds.

## 2 Materials and methods

### 2.1 Ethics approval statement

All experimental procedures involving animals were conducted in accordance with the guidelines recommended by the Administration of Affairs Concerning Experimental Animals (Ministry of Science and Technology, China, revised 2004). The experimental protocol was reviewed and approved by the Animal Research and Technology Centre of Northwest A&F University (Yangling, Shaanxi, China). The study adhered to ethical standards for the use of animals in research.

### 2.2 Animal, study design, and sample collection

This study involved a total of 1,529 healthy lactating Holstein cows from a commercial dairy farm in Ningxia, China. A subset of 219 multiparous mid-lactation Holstein cows with five or more parities, with a mean parity of 5.42 ± 0.77 (mean ± standard deviation) and mean [DIM of 148.19 ± 41.09 (mean ± standard deviation), was selected]. Within one week before the experiment began, based on previous research (Edmonson et al., 1989), experienced farm staff assessed each cow’s BCS using visual and tactile evaluations, scoring on a scale from 1 to 5 with 0.25 increments.

All cows were fed the same diet, consisting of a concentrate-to-forage ratio of 54:46 on a dry matter basis (Supplementary Table S1). The cows were fed and milked three times daily at 06:00, 14:00, and 22:00, with ad libitum access to feed and water, under the same management conditions.

The feed intake of each cow was recorded over seven consecutive days by manually measuring the initial feed weight and the remaining feed weight after each feeding period. MY was recorded continuously over 7 days, and on the seventh day, milk samples were collected in a 4:3:3 volume ratio corresponding to each milking time. The milk components, including MF, MP, ML, MSNF, MTS, MFP, MD, EC, and Ash, were analyzed using a spectrophotometer (Foss-4000; Foss Electric A/S, Hillerød, Denmark). Milk protein/fat content was multiplied by the MY recorded on the seventh day to calculate the MFY/MPY. The FE was calculated using 3.5% FCM and DMI. Cows were selected based on the absence of a history of reproductive disorders, no recorded diseases in the past 3 months, and a historical 305-day milk yield consistently exceeding 12,000 kg. Based on milk performances, 10 LH cows (parity = 5.80 ± 0.79, DIM = 128.90 ± 9.32, BCS = 3.33 ± 0.46; mean ± standard deviation) and 10 LL cows (parity = 6.1 ± 0.74, DIM = 132 ± 4.32, BCS = 3.28 ± 0.42; mean ± standard deviation) were selected for further analysis. The MY, MFY, and MPY values of LH cows were 55.32 ± 3.14 kg/day, 1.85 ± 0.11 kg/day, and 2.08 ± 0.21 kg/day (mean ± standard deviation), respectively, while those of LL cows were 38.86 ± 3.75 kg/day, 1.43 ± 0.21 kg/day, and 1.28 ± 0.18 kg/day (mean ± standard deviation) respectively. On the morning of the seventh day, before feeding, rumen contents were collected via oral tubing and filtered through four layers of sterile cheesecloth, and rectum contents were manually collected using sterile gloves, followed by 16S rRNA gene sequencing analysis, SCFAs analysis and rumen pH (RpH) measurement (Leici PHS-3C; Shanghai INESA Scientific Instrument Co., Ltd., Shanghai, China). Blood samples from all cows were collected from the tail vein in tubes without anticoagulants, and then centrifuged at 3,500 × g for 15 min at 4°C (Centrifuge 5810R, Eppendorf, Germany) to separate serum for biochemical parameter analysis.

### 2.3 Plasma parameter measurement

The concentrations of TP, ALB, GLB, ALT, AST, ALP, TBA, γ-GGT, BUN, CRE, LDH, GLU, TC, TG, HDL-C, LDL-C, IgG, IgA, SOD, GPx, T-AOC, T-GSH, and CAT in plasma were measured using an AutoAnalyzer KHB-1280 instrument (Shanhai Keshun Science and Technology Co., Ltd., Shanghai, China) with commercial kits (Beijing Jinhai Keyu Biotechnology Development Co., Ltd., Beijing, China). In addition, NEFA, BHB, HPT and SAA were analyzed using commercial enzyme-linked immunosorbent assay (ELISA) kits from Shanghai Keshun Bioengineering Institute (Shanghai, China). TNF-α, IL-1β, IL-6, IL-10, and MDA, #A003-1-2were analyzed with commercial ELISA kits from Nanjing Jiancheng Bioengineering Institute (Nanjing, China). PCwas measured using commercial ELISA kits from Beijing Solarbio Science & Technology Co., Ltd. (Beijing, China). All assays were conducted according to the manufacturer’s instructions. All samples were measured in triplicate, and the average value was calculated. All reagent kit catalogue numbers are listed in Supplementary Table S15.

### 2.4 Determination of SCFAs concentrations in ruminal fluid

Based on previous studies (Wang et al., 2023a; Wang et al., 2023b), SCFAs were determined using gas chromatography (Agilent 7820A, Santa Clara, CA, United States) with a capillary column (AE-FFAP, 30 m × 0.25 mm × 0.33 μm; ATECH Technologies Co., Lanzhou, China). In brief, rumen fluid samples were centrifuged at 12,000 × g for 10 min at 4°C. Three milliliters of the supernatant was mixed with 400 μL of 25% meta-phosphoric acid (w/v). For fecal samples, approximately 1.0 g of the sample was added to 3.0 mL distilled water and vortexed vigorously, then centrifuged at 12,000 × g for 15 min at 4°C. Two milliliters of the supernatant was then mixed with 400 μL of 25% meta-phosphoric acid (w/v). The mixture was placed at 4°C for 4 h and subsequently centrifuged at 16,000 × g for 10 min at 4°C. Then, 200 μL of caprylic acid (10 g/L) was added to 200 μL aliquots of the supernatant, and the mixture was filtered through a 0.45 μm filter. The injector and detector temperatures were set to 200°C and 250°C, respectively. The column temperature was increased from 45°C to 150°C at a rate of 20°C/min and held at 150°C for 5 min. SCFAs, including acetate (Ac), propionate (Pr), isobutyrate (iBu), butyrate (Bu), isovalerate (iVal), valerate (Val), 4-Methylvalerate (4MVal) and hexanoate (Hex), were quantitatively analyzed. In this method, the internal standard used was 2-ethylbutyrate, and the other standards included acetate, propionate, isobutyrate, butyrate, isovalerate, valerate, 4-Methylvalerate, and hexanoate. The concentration of each SCFA was quantified by constructing a standard curve using standard solutions with concentrations of 25, 50, 100, 200, 400, and 800 μM, and the total SCFA concentration was calculated by summing the concentrations of all individual SCFAs.

### 2.5 DNA extraction, 16S rRNA sequencing, and data processing for microbiota

Based on the instructions provided with the FastPure Stool DNA Isolation Kit (MJYH, Shanghai, China), total genomic DNA of the microbial community was extracted from rumen fluid and fecal samples. The integrity of the extracted genomic DNA was assessed using 1% agarose gel electrophoresis, and DNA concentration and purity were determined with a NanoDrop2000 (Thermo Scientific, United States).

Using the extracted DNA as the template, the V3–V4 hypervariable regions of the 16S rRNA gene were PCR-amplified with a forward primer 338F (5′-ACTCCTACGGGAGGCAGCAG-3′) carrying a barcode sequence and a reverse primer 806R (5′-GGACTACHVGGGTWTCTAAT-3′) (Liu et al., 2016). The PCR reaction mixture consisted of 4 μL of 5 × TransStart FastPfu buffer, 2 μL of 2.5 mM dNTPs, 0.8 μL of the forward primer (5 μM), 0.8 μL of the reverse primer (5 μM), 0.4 μL of TransStart FastPfu DNA polymerase, 10 ng of template DNA, and ddH₂O to a final volume of 20 μL. The amplification program was as follows: initial denaturation at 95°C for 3 min; 27 cycles of 95°C for 30 s (denaturation), 55°C for 30 s (annealing), and 72°C for 30 s (extension); followed by a final extension at 72°C for 10 min, with samples subsequently stored at 4°C (PCR instrument: ABI GeneAmp^®^ 9700). The PCR products were recovered using 2% agarose gel electrophoresis and purified with the DNA Gel Recovery Purification Kit (PCR Clean-Up Kit, China Yuhua). Quantification of the purified products was performed using a Qubit 4.0 (Thermo Fisher Scientific, United States). Subsequently, the purified PCR products underwent library construction using the NEXTFLEX Rapid DNA-Seq Kit, which involved: (1) Adapter ligation. (2) Magnetic bead screening to remove adapter self-ligation fragments. (3) PCR amplification for library template enrichment. (4) Magnetic bead recovery of the PCR products to obtain the final library. Sequencing was conducted on the Illumina NextSeq2000 platform (Shanghai Meiji Biomedical Technology Co., Ltd.).

The paired-end raw sequencing reads were quality controlled using fastp (Chen et al., 2018) (https://github.com/OpenGene/fastp, version 0.19.6) and merged using FLASH (Magoč and Salzberg, 2011) (http://www.cbcb.umd.edu/software/flash, version 1.2.11) with the following steps: (1) Filter out bases at the read ends with quality values below 20 using a 50 bp sliding window; if the average quality in the window is below 20, trim the bases from the beginning of the window; remove reads shorter than 50 bp after quality control, and discard reads containing ambiguous “N” bases. (2) Merge paired-end reads into a single sequence based on the overlap between the paired-end reads, with a minimum overlap length of 10 bp. (3) Allow a maximum mismatch rate of 0.2 in the overlap region of the merged sequence and filter out sequences that do not meet this criterion. (4) Demultiplex the samples and adjust sequence orientations according to the barcode and primer sequences at both ends, allowing 0 mismatches for barcodes and up to 2 mismatches for primers. Using the default parameters, the quality-controlled and merged sequences were denoised using the DADA2 (Edgar, 2013) plugin within the Qiime2 pipeline (Barberán et al., 2012). The sequences resulting from DADA2 denoising are commonly referred to as ASVs (amplicon sequence variants). All sequences annotated as chloroplast or mitochondrial were removed from all samples. To minimize the impact of sequencing depth on subsequent alpha and beta diversity analyses, all samples were rarefied to 20,000 sequences, with the average Good’s coverage per sample remaining at 99.09%. Taxonomic classification of the ASVs was performed using a Naive Bayes classifier in QIIME2, based on the SILVA 16S rRNA gene database (v138) (Quast et al., 2013). Functional prediction of 16S data was conducted using PICRUSt2 (Segata et al., 2011) (version 2.2.0). Additionally, using BugBase to assess microbial community characteristics (Ward et al., 2017).

### 2.6 Biomarker selection for LH and LL cows using Random Forest

To differentiate between LH cows and LL cows, a random forest classification model was constructed using the RandomForest package in R. Bacterial relative abundance from the rumen and rectum, concentrations of SCFAs and serum biochemical parameters were included as independent variables in the model. The classification performance was validated using the area under the receiver operating characteristic (ROC) curve (area under the curve, AUC). Variable importance was assessed using the mean decrease accuracy (MDA) metric to identify biomarkers that significantly contributed to the classification.

### 2.7 Statistical analysis

Physiological parameters, serum parameters, rumen and rectum SCFA concentrations, and bacterial abundance and functional data were analyzed using SAS software (version 9.4, SAS Institute Inc., Cary, NC, United States). A mixed procedure was used as follows:

$$Y_{ijk}=\mu +G_{i}+KC+e_{ijk}$$

where $Y_{ijk}$ is the dependent variable, μ is the overall mean, $G_{i}$ is the effect of the ith group, C is the vector of the fixed covariates, consisting of parity, DIM and BCS. $e_{ijk}$ is the residual error. Independent sample *t*-test was used to compare differences between groups. The Wilcoxon rank-sum test was performed to compare the inter-group α diversity of rumen and rectum microbial communities, with significance determined by false discovery rate (FDR)-adjusted *p*-values <0.05. Principal coordinate analysis (PCoA) based on the Bray–Curtis distance algorithm was used to assess β diversity, and inter-group differences were tested using the adonis method (a permutational multivariate analysis of variance, PERMANOVA). Linear Discriminant Analysis Effect Size (LEfSe) was used to compare taxonomic, functional and KEGG enzymes; significance was determined with a *p*-value <0.05 and LDA >2. Correlations between phenotypes were assessed using Mantel’s test. Distance matrices for bacterial communities and phenotypes were calculated using the Bray–Curtis distance algorithm. Spearman’s rank correlation was employed to analyze the correlations between these matrices, with significance determined by Mantel’s *p*-values and Spearman’s *p*-values <0.05. Additionally, Spearman’s correlation analysis was conducted to evaluate the associations between the relative abundances of differential bacteria and the differential serum phenotypes and SCFAs, considering correlations significant when *p*-values <0.05 and Spearman’s |*r*| >0.50. Spearman correlation coefficients were calculated using the Hmisc package (version 4.6.0) in R to analyze the genus-level association networks in LH and LL cows. Significant correlations between genera (*p*-values <0.05 and Spearman’s |*r*| >0.60) were visualized using Cytoscape (version 3.8.2) (Shannon et al., 2003). The bacterial interaction networks were further modularized using the Leiden clustering algorithm (Traag et al., 2019). Spearman’s rank correlations were established between milk production phenotypes, differential serological parameters, rumen and rectum differential SCFAs, KEGG enzymes, and bacterial relative abundance of the two groups of cows (Spearman |*r*| >0.50 and *p*-value <0.05). Correlation and Mantel test heatmaps were visualized using R (v4.3.1). The Mantel test was performed using the vegan package (v2.6.6.1), and correlation and Mantel test heatmaps were visualized using ComplexHeatmap package (v2.21.0). The multiplex network was visualized using Cytoscape software.

## 3 Results

### 3.1 Differences in microbial composition of the rumen and rectum between LH and LL groups

**Figure 1:**
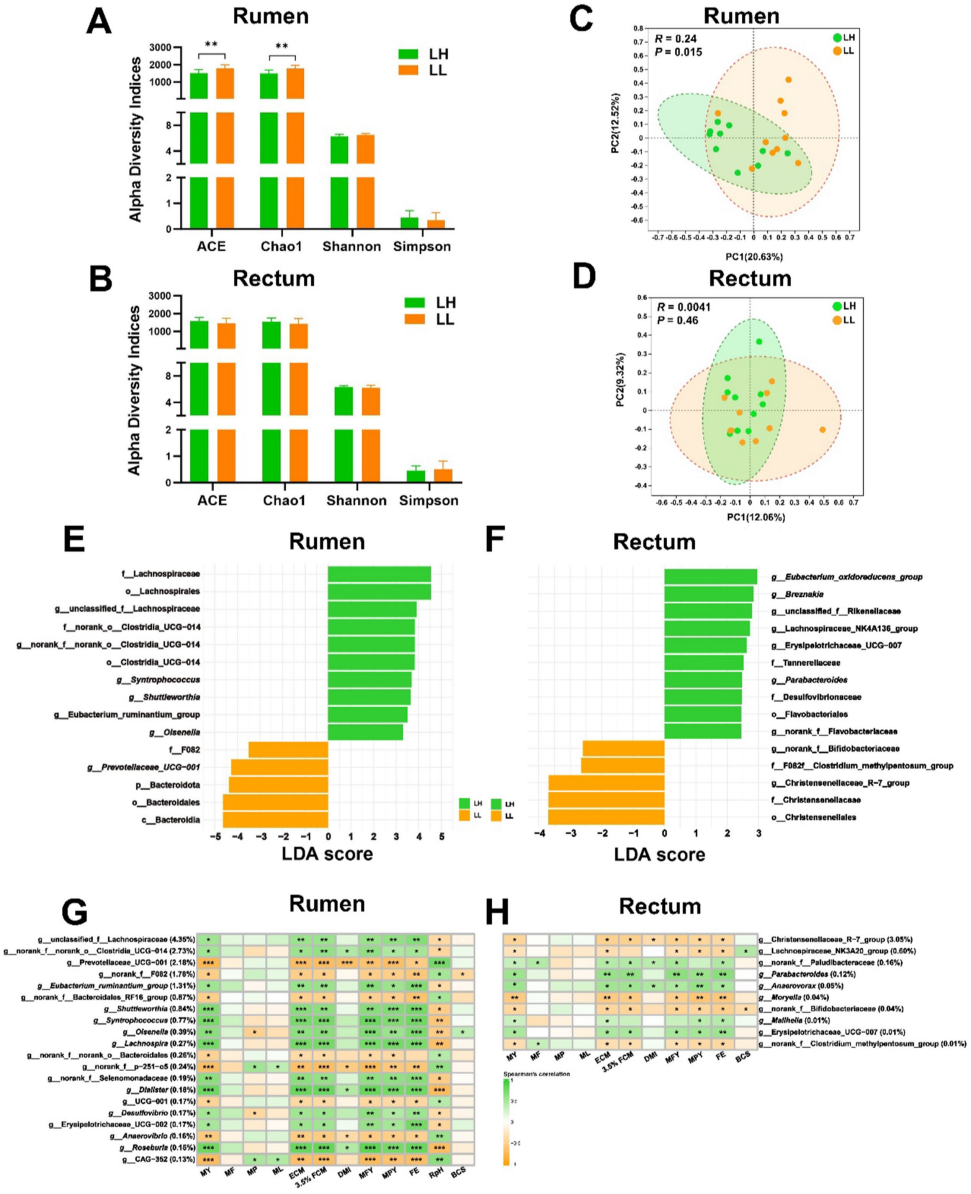
Comparison of microbiota diversity and structure in the rumen and rectum of LH and LL groups. **(A,B)** Alpha diversity indices of rumen and rectum microbiota. Error bars represent mean ± SEM. ^**^p < 0.01. **(C,D)** The PCoA of rumen and rectum microbiota at the ASV level were based on the Bray–Curtis dissimilarity. Dissimilarity was analyzed using ANOSIM statistical tests with 999 permutations. **(E,F)** The LEfSe bar plots show differentially abundant bacterial taxa between LH and LL groups in rumen and rectum microbial community. The significance threshold was set at LDA >2 and *p* < 0.05. **(G,H)** Heatmaps show the association between rectum and rumen bacterial genera (average relative abundance > 0.1 and 0.01%) and the production and physiological parameters (Spearman’s correlation). ^*^p < 0.05, ^**^p < 0.01, and ^***^p < 0.001.

The richness of the rumen microbiota in LH cows was significantly lower than that in LL cows (*p* < 0.05), while diversity and evenness showed no significant differences (Figure 1A). The alpha diversity of the rectum microbiota also did not differ significantly between the two groups (Figure 1B). At the ASV level in the rumen, the Chao1, ACE, and Shannon indices were negatively correlated with MY, while the Simpson index showed a positive correlation with MY (*p* < 0.05). However, no significant correlations were observed between the alpha diversity of the rectum microbiota and production phenotypes at any taxonomic level (Supplementary Table S2). PCoA revealed that the rumen bacterial community compositions differed significantly between LH and LL cows (*p* < 0.05); however, no significant differences were observed in rectum microbiota community between the two groups (Figures 1C,D). At the phylum level, only Bacteroidota, Fibrobacterota, and Verrucomicrobiota exhibited significant differences in the rumen bacterial composition between LH and LL cows (*p* < 0.05), with LH cows exhibiting significantly lower levels than LL cows. However, there were no differences in rectum bacterial composition at the phylum level between the two groups of cows (Supplementary Figure S1A and Supplementary Tables S3, S4). At the genus level, there were 43 differentially abundant genera in the rumen bacteria between the LH and LL groups, and 14 differentially abundant genera in the rectum bacteria between the two groups (Supplementary Figure S1B and Supplementary Tables S3, S4). LEfSe analysis identified enriched bacterial genera (LDA >2), including unclassified_f__Lachnospiraceae, norank_f__norank_o__Clostridia_UCG-014, *Shuttleworthia*, *Syntrophococcus*, *Eubacterium_ruminantium_group*, and *Olsenella* in the rumen microbiota of the LH group (Figure 1E and Supplementary Figure S1A). Bacterial genera (LDA >2) such as *Eubacterium_oxidoreducens_group*, *Breznakia*, *Parabacteroides*, and norank_f__Flavobacteriaceae were enriched in the rectum samples from the LH group (Figure 1F and Supplementary Figure S2B). In the rumen, the most abundant genera included unclassified_f__Lachnospiraceae, norank_f__norank_o__Clostridia_UCG-014, *Eubacterium_ruminantium_group*, *Shuttleworthia*, *Syntrophococcus*, *Olsenella*, *Lachnospira*, norank_f__Selenomonadaceae, *Dialister*, *Desulfovibrio*, Erysipelotrichaceae_UCG-002, and *Roseburia* (relative abundance >0.1%; *p* < 0.05) showed a positive correlation with milk production phenotypes, including MY, ECM, 3.5% FCM, MFY, MPY, and FE, while Prevotellaceae_UCG-001, norank_f__F082, norank_f__Bacteroidales_RF16_group, norank_f__norank_o_Bacteroidales, norank_f__p-251-o5, UCG-001, *Anaerovibrio*, and g__CAG-352 (relative abundance >0.1%; *p* < 0.05) were negatively correlated with milk production phenotypes (Figure 1G). In the rectum, genera such as norank_f__Paludibacteraceae, *Parabacteroides*, *Anaerovorax*, *Mailhella*, and Erysipelotrichaceae_UCG-007 (relative abundance >0.01%; *p* < 0.05) showed a positive correlation with milk production phenotypes, while the Christensenellaceae_R-7_group, Lachnospiraceae_NK3A20_group, *Moryella*, norank_f__Bifidobacteriaceae, and norank_f__Clostridium_methylpentosum_group (relative abundance >0.01%; *p* < 0.05) were negatively correlated with milk production phenotypes (Figure 1H).

### 3.2 Distinct rumen and rectum bacterial phenotypes in LH vs. LL cows

**Figure 2:**
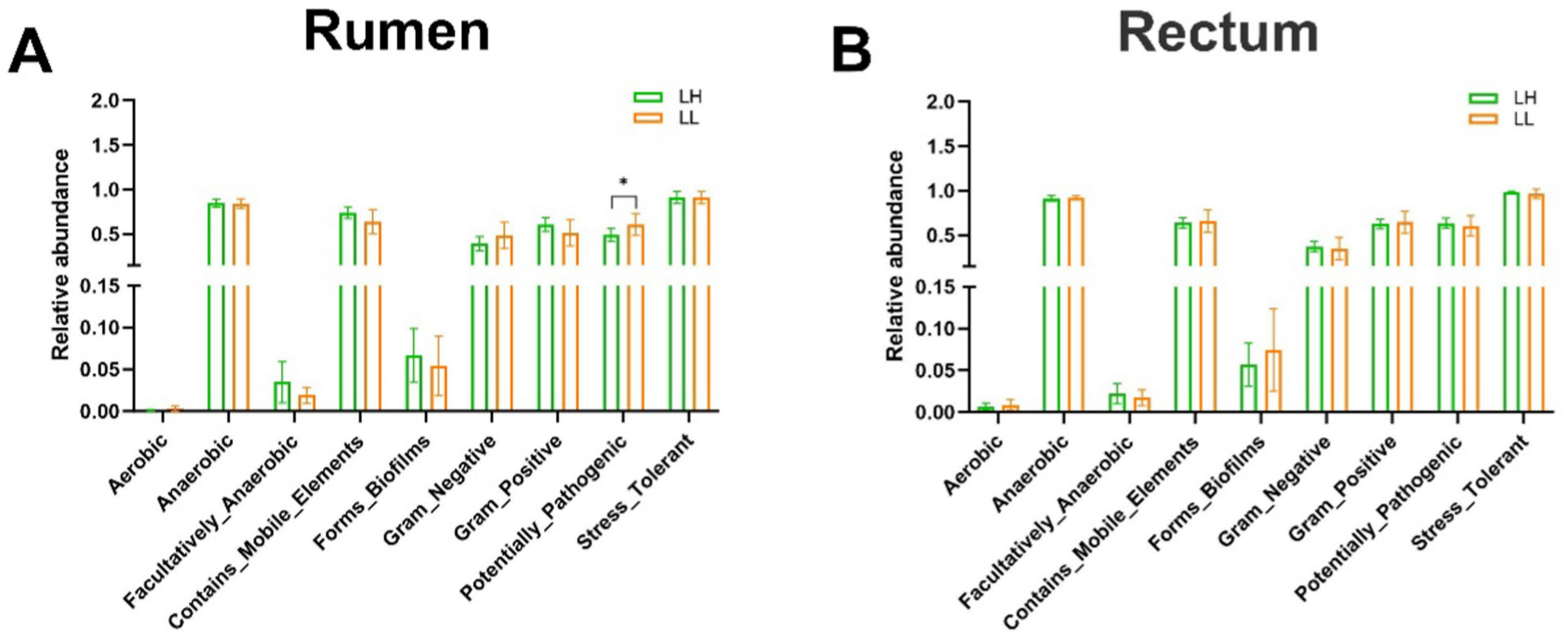
Differences in the BugBase-predicted microbial phenotypes between LH and LL groups. **(A,B)** Prediction of nine microbial phenotypes in the rumen and rectum of LH and LL groups using BugBase phenotype prediction. The bars represent mean ± SEM. ^*^p < 0.05.

BugBase predictive analysis of nine bacterial phenotypes in the rumen and rectum of both groups revealed that the pathogenic potential of rumen bacteria in LH cows was lower than in LL cows (*p* < 0.05), while no significant differences were observed in the rectum bacterial phenotypes (Figures 2A,B). These results indicate that the rumen bacterial community in LH cows exhibits lower pathogenic potential.

### 3.3 Different phenotypes and their association with bacteria

**Figure 3:**
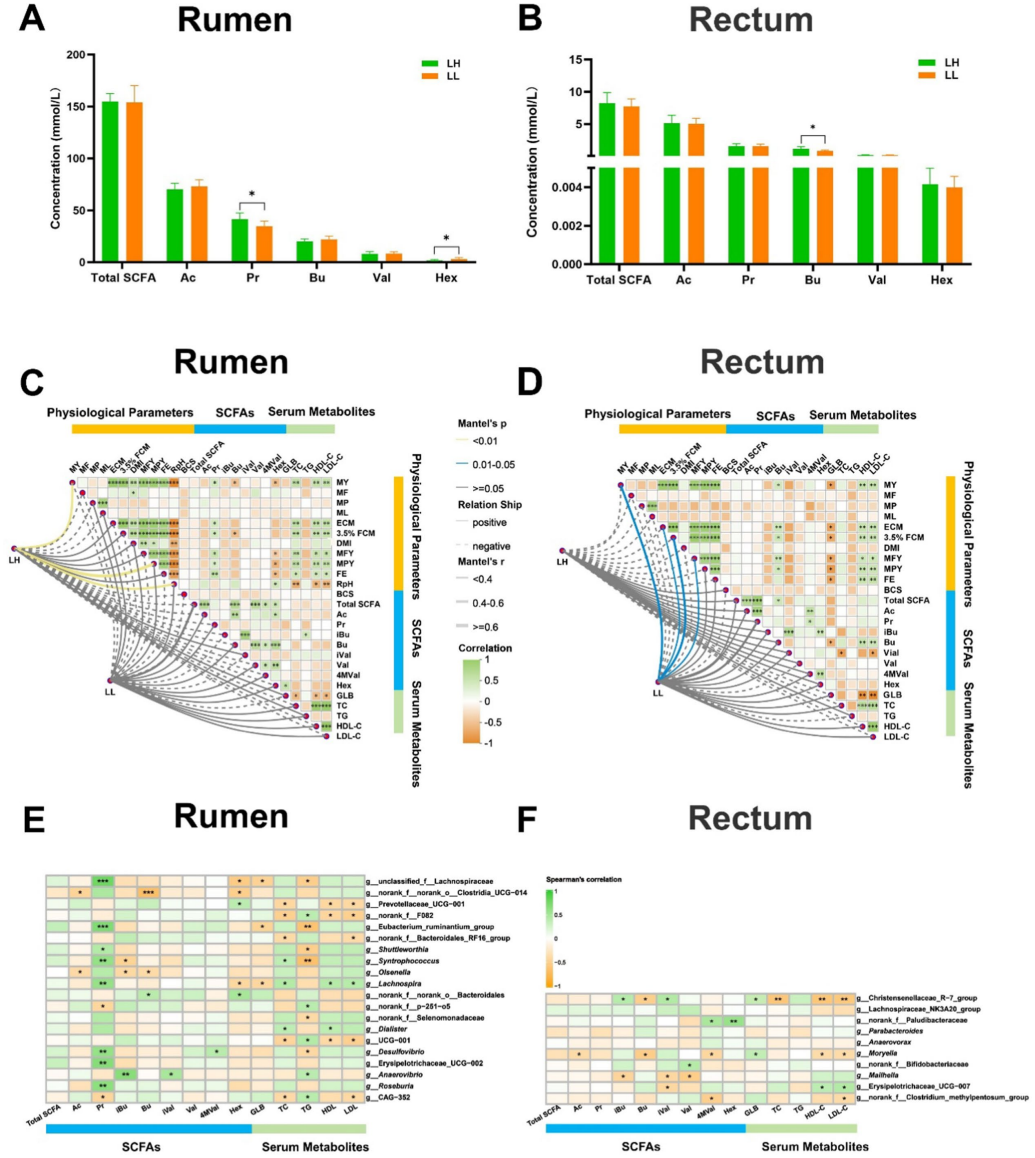
Correlation between rumen and rectum SCFAs, differential serum parameters and MY-related microbiota. **(A,B)** Concentrations of SCFAs in the rumen and rectum of the LH and LL groups. The bars represent mean ± SEM. ^*^p < 0.05. **(C,D)** Correlation matrix among physiological parameters, SCFA concentrations in rumen and rectum, and serum metabolites in the LH and LL groups were calculated using Mantel’s test. The distance matrix for clinical factors was computed based on the Bray–Curtis algorithm, while Spearman’s correlation coefficients were used to evaluate associations. ^*^p < 0.05, ^**^p < 0.01, and ^***^p < 0.001. **(E,F)** Spearman’s rank correlation heatmaps between MY-related bacteria and SCFAs in rumen and rectum. The color gradient represents the values of correlation coefficients. ^*^p < 0.05, ^**^p < 0.01, and ^***^p < 0.001.

The milk composition and DMI showed no significant differences between LH and LL cows. However, LH cows exhibited significantly higher MY, MFY, MPY, ECM, 3.5% FCM, FE, 305-d MY, historical average 305-d MY, and lower RpH compared with LL cows (*p* < 0.05; Table 1). In the serum of LH cows, TC, HDL-C, and LDL-C were significantly higher, while GLB and TG were significantly lower than in LL cows (*p* < 0.05; Table 2). Compared to the LL group (*p* < 0.05), LH cows showed higher concentrations of Pr and lower concentrations of Hex in the rumen, along with higher Bu concentrations in the rectum; acetate and other SCFAs did not show significant differences between the two groups in rumen and rectum (Figures 3A,B and Supplementary Table S5). Results of the Mantel test based on the rumen bacteria matrix indicated that rumen Bu and Hex concentrations were negatively correlated with MY (*p* < 0.05), while rumen Pr, serum TC, HDL-C, and LDL-C concentrations were positively correlated with milk production phenotypess; rumen acetate and other SCFAs were not found to be associated with any milk production phenotypes (*p* < 0.05; Figure 3C). The Mantel test results based on the rectum bacteria matrix indicated a positive correlation between rectum Bu concentration and milk production phenotypes, a negative correlation between serum GLB concentration and milk production phenotypes, and positive correlations between HDL-C, LDL-C, and milk production phenotypes; rectum acetate and other SCFAs were also not found to be associated with any milk production phenotypes (*p* < 0.05; Figure 3D). In the rumen, the relative abundances of MY-associated bacteria unclassified_f__Lachnospiraceae, *Eubacterium_ruminantium_group*, *Shuttleworthia Syntrophococcus*, *Lachnospira*, *Desulfovibrio*, Erysipelotrichaceae_UCG-002, and *Roseburia* were positively correlated with Pr concentrations (*p* < 0.05; Figure 3E). In the rectum, the relative abundance of MY-associated bacteria of the Christensenellaceae_R-7_group and *Moryella* showed negative correlations with Bu concentration (*p* < 0.05; Figure 3F).

**Table 1:** Physiological parameters of LH and LL cows.

	Mean			
	LH	LL		
^a^Performance	(*n* = 10)	(*n* = 10)	^b^SEM	*p*-value
MF (%)	3.76	3.75	0.15	0.82
MP (%)	3.34	3.36	0.09	0.63
ML (%)	4.72	4.75	0.14	0.84
MSNF (%)	8.80	8.89	0.24	0.69
MTS (%)	12.52	12.65	0.27	0.66
MFP (°C)	−0.54	−0.54	0.02	0.90
MD (g/cm^3^)	1.03	1.03	0.001	0.86
EC (mS/cm)	5.04	4.97	0.11	0.52
Ash (%)	0.76	0.78	0.02	0.31
MY (kg/day)	55.32	38.86	1.45	< 0.001
MFY (kg/day)	2.08	1.43	0.10	< 0.001
MPY (kg/day)	1.84	1.28	0.07	< 0.001
ECM (kg/day)	58.39	40.24	1.37	<0.001
3.5% FCM (kg/day)	43.74	30.07	1.98	<0.001
DMI (kg/day)	25.22	24.03	0.70	0.10
FE (kg/kg)	1.74	1.25	0.05	<0.001
305-d MY (kg/day)	50.71	38.07	1.53	<0.001
Historical average 305-d MY (kg/day)	45.20	40.14	1.98	<0.001
BCS	3.33	3.28	0.20	0.80
Parity	5.80	6.10	0.48	0.39
DIM (days)	128.90	134.00	3.24	0.14
RpH	5.88	6.20	0.07	<0.001

a SEM, stand error of the mean. *p*-values between LH and LL were calculated using *t*-test.

b MF, milk fat; MP, milk protein; ML, milk lactose; MSNF, milk solid-not-fat; MTS, milk total solids; MFP, milk freezing point; MD, milk density; EC, electrical conductivity; Ash, ash content; MY, milk yield; MFY, milk fat yield; MPY, milk protein yield; ECM, energy corrected milk; 3.5% FCM, 3.5% fat-corrected milk; DMI, dry matter intake. DMI was defined as the total dry matter intake from all feed components; FE, feed efficiency = MY/DMI; BCS, body condition score; DIM, days in milk; RpH, rumen pH.

**Table 2:** Serum biochemical of LH and LL cows.

	Mean			
	LH	LL		
^a^Performance	(*n* = 10)	(*n* = 10)	^b^SEM	*p*-value
Serum biochemical parameters				
TP (g/L)	71.38	74.46	2.88	0.18
ALB (g/L)	44.26	43.42	2.33	0.63
GLB (g/L)	27.24	31.01	0.14	<0.05
ALB/GLB	1.64	1.43	0.17	0.09
ALT (U/L)	43.26	39.84	2.31	0.05
AST (U/L)	84.84	86.99	6.27	0.64
ALP (U/L)	230.85	218.49	20.26	0.40
TBA (μmol/L)	4.98	5.64	0.64	0.16
γ-GGT (U/L)	31.60	32.04	4.77	0.90
BUN (mmol/L)	5.57	5.22	0.49	0.34
CRE (μmol/L)	77.22	79.23	5.27	0.60
LDH (U/L)	672.94	716.85	31.22	0.18
GLU (mmol/L)	4.13	4.50	0.26	0.18
TC (mmol/L)	4.63	3.89	0.26	<0.05
TG (mmol/L)	0.48	0.53	0.02	<0.05
HDL-C (mmol/L)	2.17	1.91	0.09	<0.01
LDL-C (mmol/L)	2.36	1.87	0.18	<0.05
NEFA (μmol/L)	388.87	458.18	48.69	0.17
BHB (μmol/L)	725.46	751.71	43.72	0.56
Oxidative stress biomarkers				
SOD (U/mL)	106.62	104.90	5.83	0.70
GSX-PX (U/mL)	60.27	59.41	1.68	0.62
T-AOC (mmol/L)	1.90	1.88	0.09	0.87
T-GSH (μmol/L)	3.68	3.58	0.10	0.30
CAT (U/mL)	5.96	5.79	0.45	0.62
MDA (μmol/L)	3.70	3.65	0.09	0.48
PC (μmol/L)	1.62	1.65	0.07	0.76
Inflammatory and immunological markers				
IL-1β (pg/mL)	8.47	8.54	0.92	0.94
IL-6 (pg/mL)	22.04	21.50	1.84	0.77
IL-10 (pg/mL)	12.17	12.04	1.06	0.90
HPT (μg/mL)	39.58	39.39	3.47	0.96
TNF-α (pg/mL)	16.41	15.46	1.31	0.48
SAA (μg/mL)	24.42	25.81	2.81	0.63
IgG (g/L)	21.94	21.74	1.04	0.85
IgA (g/L)	0.81	0.78	0.07	0.73

a SEM, stand error of the mean. *p*-values between LH and LL were calculated using *t*-test.

b TP, total protein; ALB, albumin; GLB; globulin; ALB/GLB, ALB/GLB rate; ALT, alanine aminotransferase; AST, aspartate aminotransferase; ALP, alkaline phosphatase; TBA, total bile acids; γ-GGT, gamma-glutamyl transferase; BUN, blood urea nitrogen; CRE, creatinine; LDH, lactate dehydrogenase; GLU, glucose; TC, total cholesterol; TG, triglycerides; HDL-C, high-density lipoprotein cholesterol; LDL-C, low-density lipoprotein cholesterol; NEFA, non-esterified fatty acids; BHB, beta-hydroxybutyrate; SOD, superoxide dismutase; GPx, glutathione peroxidase; T-AOC, total antioxidant capacity; T-GSH, total glutathione; CAT, catalase; MDA, malondialdehyde; PC, protein carbonyl; IL-1β, interleukin-1 beta; IL-6, interleukin-6; IL-10, interleukin-10; HPT, haptoglobin; TNF-α, tumor necrosis factor-alpha; SAA, serum amyloid A; IgG, immunoglobulin G; IgA, immunoglobulin A.

### 3.4 Microbial co-occurrence patterns and their association with phenotypes

**Figure 4:**
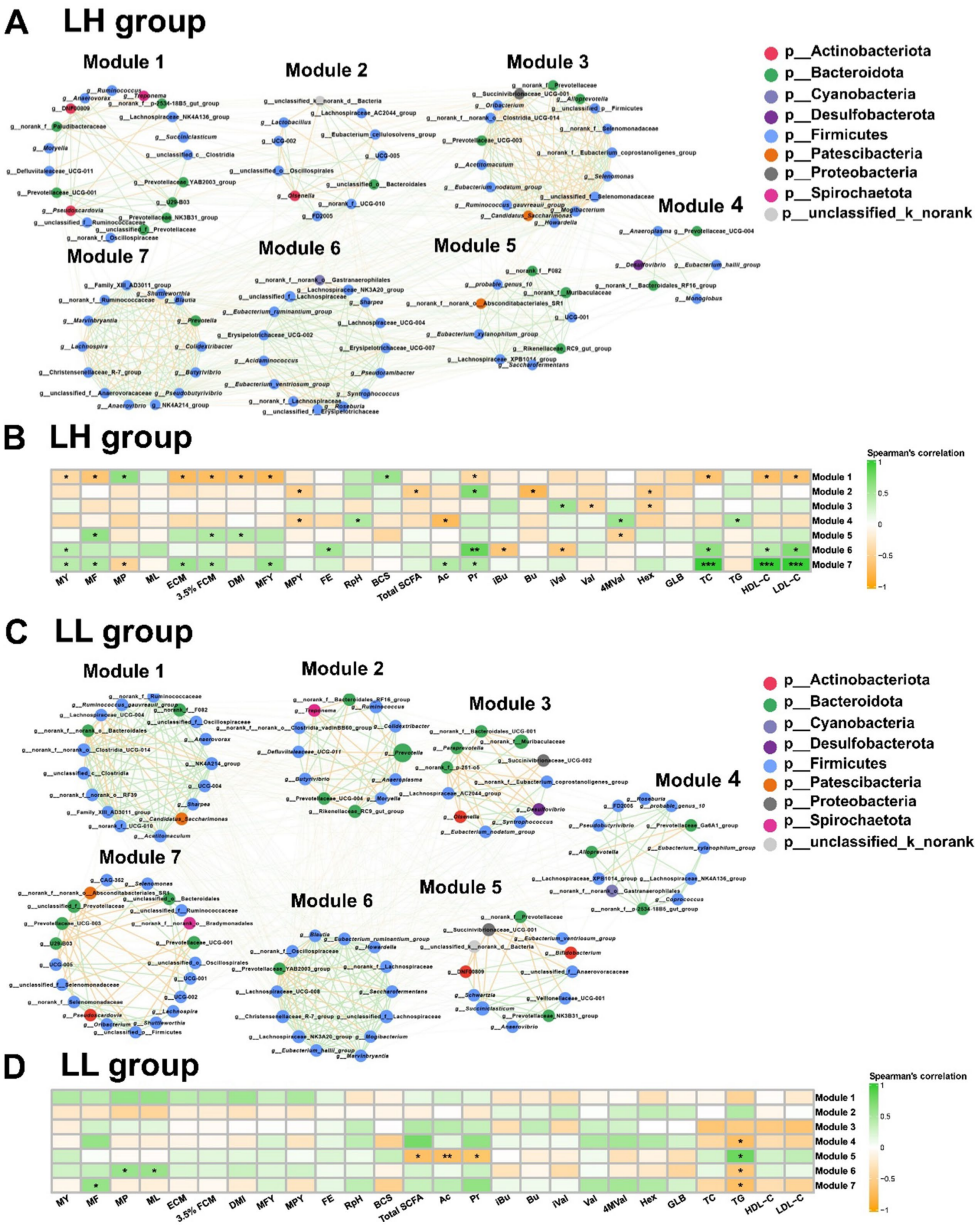
Co-occurrence networks and correlation analysis of rumen microbiota in LH and LL cows. **(A,C)** Co-occurrence network of rumen bacteria at the genus level, with bacterial genera assigned based on their network roles in LH and LL cows (*n* = 10 per group). Nodes represent bacterial genera, with node size indicating the relative abundance of each genus. The color of the edges between nodes indicates positive (green) or negative (yellow) correlations (Spearman’s |*r*| >0.60 and *p* < 0.05). The thickness of the edges represents the magnitude of Spearman’s |*r*|. **(B,D)** Correlation analysis on the rumen microbiota modules and phenotypes in LH and LL group. The color gradient represents the values of correlation coefficients (Spearman’s correlation). ^*^p < 0.05, ^**^p < 0.01, and ^***^p < 0.001.

We used microbial co-occurrence networks to identify key microbial populations in LH and LL cows. In the rumen bacteria of LH cows, Module 1 was centered on the Prevotellaceae_UCG-001, which was directly connected to 25 bacterial genera and had the highest closeness centrality, thereby establishing it as the key pathway node. Module 1 was negatively correlated with milk production phenotypes, as well as rumen Pr, serum TC, HDL-C, and LDL-C concentrations (*p* < 0.05). In Module 6, *Syntrophococcus* exhibited the highest betweenness centrality and was connected with unclassified_f__Lachnospiraceae, *Roseburia*, *Eubacterium_ruminantium_group*, and Erysipelotrichaceae_UCG-002, all of which were enriched in LH cows. Module 6 was positively correlated with MY, FE, rumen Pr, and serum TC, HDL-C, and LDL-C concentrations (*p* < 0.05). In Module 7, *Lachnospira* and *Shuttleworthia* showed the highest betweenness centrality and closeness centrality, connecting directly with 25 and 29 bacterial genera, respectively, thus making them the core nodes of this module (Figure 4A and Supplementary Table S6). This module, centered on *Lachnospira* and *Shuttleworthia*, was positively correlated with MY, rumen Ac, Pr and serum TC, HDL-C, and LDL-C concentrations (*p* < 0.05; Figure 4B). In contrast, none of the modules showed a correlation between the rumen bacteria of LL cows and MY. However, in Module 5, *Anaerovibrio* exhibited the highest betweenness centrality and closeness centrality, serving as the core node (Figure 4C and Supplementary Table S6). This module was negatively correlated with total SCFAs, Ac, and Pr concentrations in the rumen (*p* < 0.05; Figure 4D).

For the rectum bacteria in LH cows, Module 1 was positively correlated with MY, rectum Bu, and Hex concentration (*p* < 0.05; (Supplementary Figures S4A,B). Although the *Eubacterium_oxidoreducens_group*, a core bacterium within this module, was not directly correlated with MY, it was enriched in LH cows, connected to 13 other genera, and had the highest betweenness centrality and closeness centrality, suggesting that it could have indirectly influenced MY by maintaining connectivity and functional cooperation within the module (Supplementary Figures S3A,B and Supplementary Table S7). In the rectum bacteria of LL cows, none of the modules showed any correlation with MY (Supplementary Figures S3C,D).

### 3.5 Differential functions of the rumen microbiome between LH and LL groups

**Figure 5:**
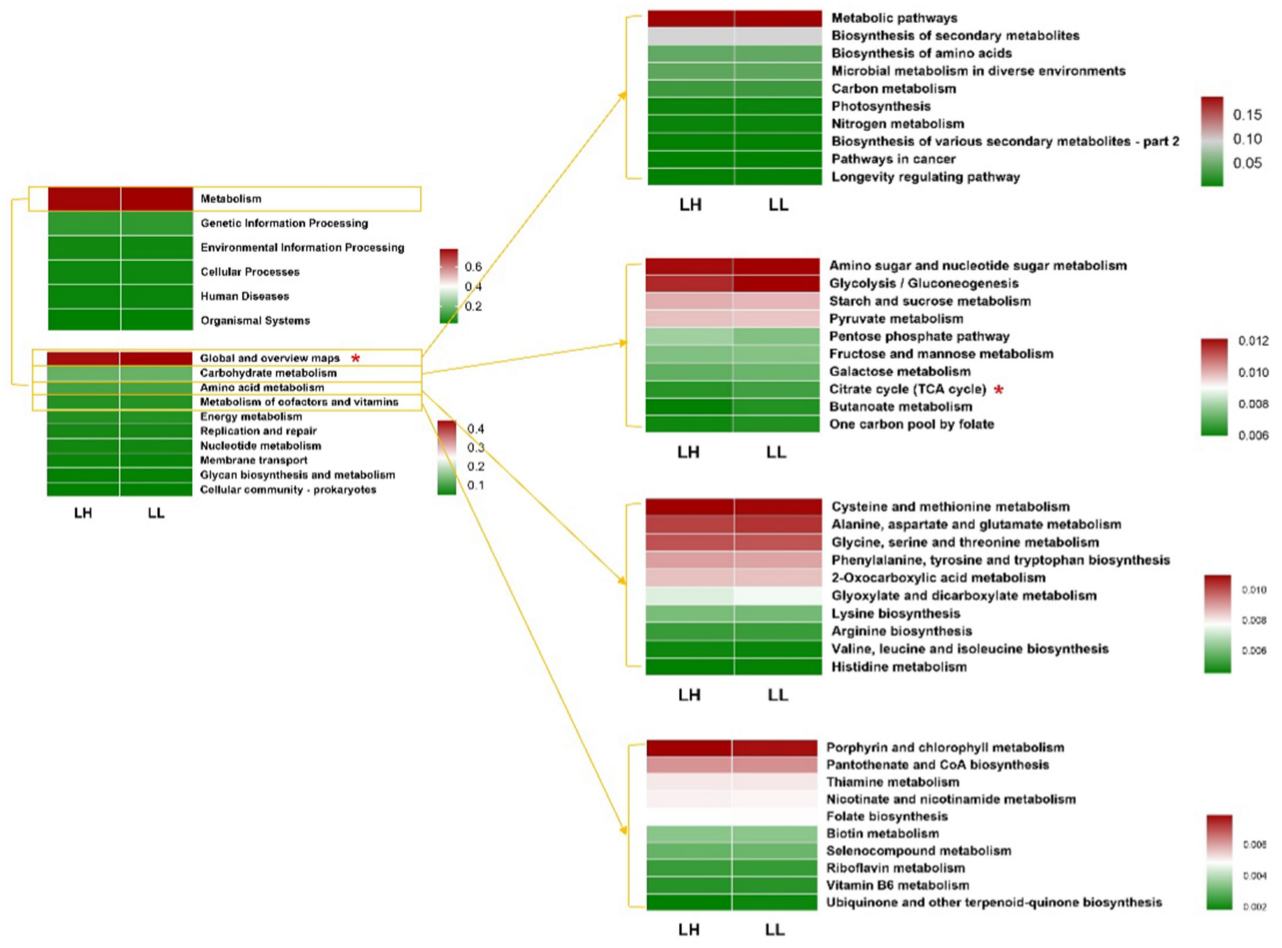
Functional predictions of rumen bacteria between LH and LL groups by PICRUSt2. Relative abundances of functional KEGG pathways at level 1, level 2, and level 3 in rumen bacteria (top 10 of global and overview map, carbohydrate metabolism, amino acid metabolism, and metabolism of cofactors and vitamins). KEGG pathways were compared using Student’s *t*-tests. ^*^p < 0.05.

Using PICRUSt2, the functional prediction of rumen microbial communities compared the KEGG pathways between the two groups of dairy cows. At KEGG pathway level 1, the rumen microbiota of dairy cows was functionally identified with Metabolism (78.22%), Genetic Information Processing (8.84%), Environmental Information Processing (4.23%), Cellular Processes (3.873%), Human Diseases (3.047%), and Organismal Systems (1.841%). No significant differences were observed between the two groups of cows at this level. At KEGG pathway levels 2 and 3, the global and overview maps and citrate cycle pathway in the rumen microbiota of LL cows were significantly higher than those of LH cows (Figure 5 and Supplementary Table S8). For the rectum microbiota of the two groups, no significant differences were detected at any KEGG pathway level (Supplementary Table S9 and Supplementary Figure S4).

**Figure 6:**
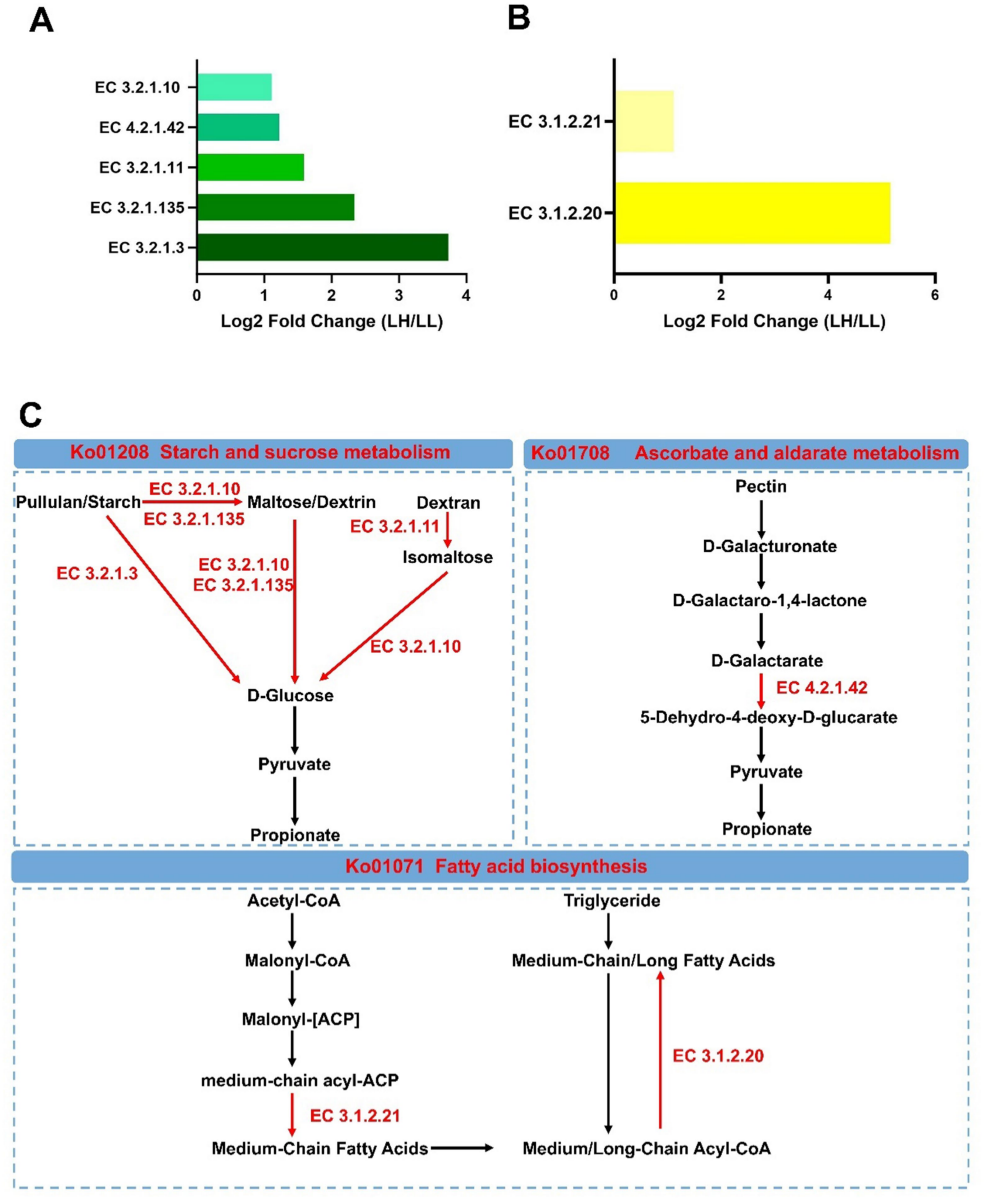
Differential rumen KEGG enzymes and metabolic pathways between LH and LL groups. **(A,B)** Significantly different rumen carbohydrate-related and lipid-related KEGG enzymes between LH and LL groups (LDA >2, *p* < 0.05). **(C)** Differential microbial metabolic pathways between LH and LL groups. Red names and arrows indicate KEGG orthology and KEGG enzymes enriched in LH cows. EC, Enzyme Commission.

Additionally, seven KEGG enzymes related to carbohydrate and fatty acid metabolism were significantly enriched in the LH group (log2 fold change >1, LDA >2 and *p* < 0.05) (Figures 6A,B). Among the KEGG enzymes involved in carbohydrate metabolism, EC 3.2.1.10 (oligo-1,6-glucosidase; involved in the hydrolysis of α-1,6 glycosidic bonds in oligosaccharides derived from starch and glycogen), EC 3.2.1.11 (dextranase; involved in the hydrolysis of α-1,6 glycosidic bonds in dextran), EC 3.2.1.3 (glucoamylase; involved in the hydrolysis of terminal α-1,4 glycosidic bonds in starch and related polysaccharides), EC 3.2.1.135 (neopullulanase; involved in the hydrolysis of pullulan by cleaving α-1,4 glycosidic bonds) and EC 4.2.1.42 (galactarate dehydratase; involved in the dehydration of galactarate to 5-dehydro-4-deoxy-D-glucarate) were more abundant in the LH group. EC 3.1.2.21 [medium-chain acyl-(acyl-carrier-protein) hydrolase; involved in hydrolyzing medium-chain acyl groups from acyl carrier protein] and EC 3.1.2.20 (acyl-CoA hydrolase; involved in hydrolyzing acyl-CoA to produce free fatty acids and coenzyme A) in the fatty acid biosynthesis pathway were more abundant in the LH group (Figure 6C). However, two KEGG enzymes, EC 1.1.1.303 (diacetyl reductase; involved in reducing diacetyl to Acetoin) and EC 1.1.1.4, (R,R)-butanediol dehydrogenase [involved in the reversible conversion between (R,R)-2,3-butanediol and acetoin], which promote the conversion of propionate to (R,R)-butane-2,3-diol, were significantly reduced in the rectums of LL cows (log2 fold change >1, LDA >2 and *p* < 0.05) (Supplementary Figures S5A,B). The mixed model analysis confirmed these findings with consistent results (*p* < 0.05) (Supplementary Table S14).

### 3.6 Relationships between microorganisms, microbial functions and phenotypes

**Figure 7:**
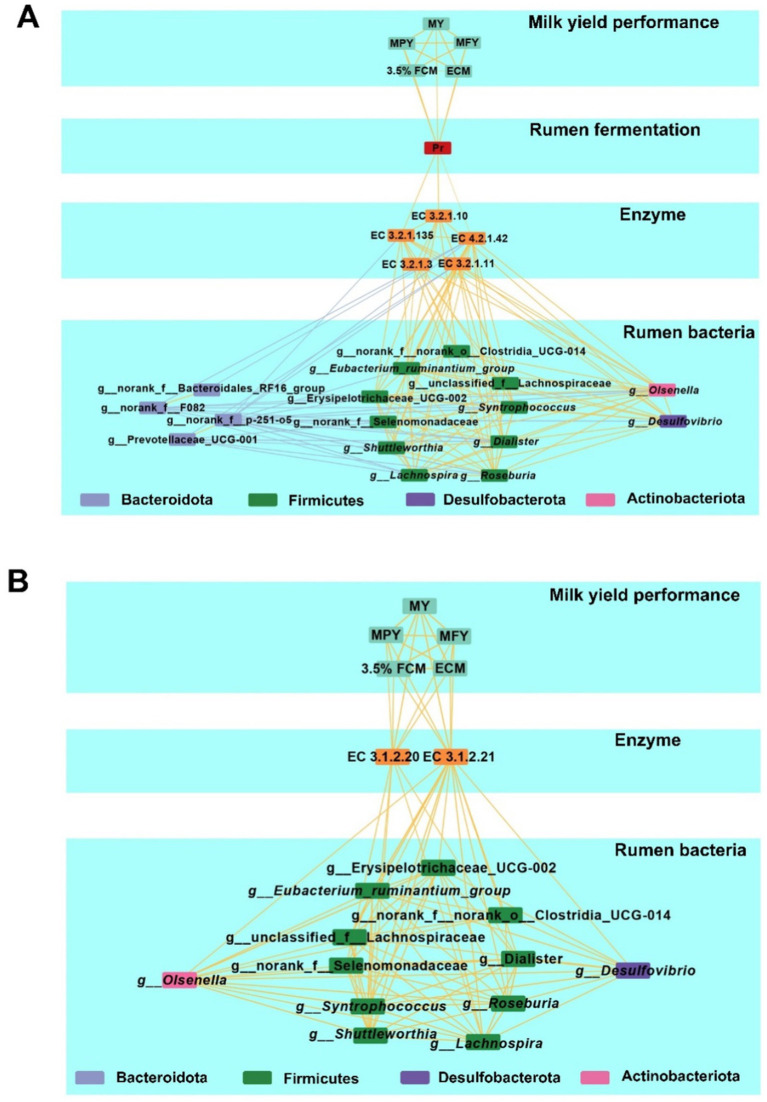
Multiplex networks showing the relationships between phenotypes. **(A)** Multiplex networks about the relationships between rumen bacteria, rumen carbohydrate-related KEGG enzymes, rumen fermentation parameter, and milk production phenotypes. **(B)** Multiplex networks showing the relationships between rumen bacteria, rumen lipid-related KEGG enzymes, and milk production phenotypes. Lines between two nodes represent the correlation, with a yellow line indicating a positive correlation and a blue line indicating a negative correlation; Line thickness represents the strength of the correlation (Spearman’s |*r*| >0.50 and *p* < 0.05).

A multi-layered network based on Spearman rank correlations was constructed to determine the relationships among MY-associated rumen bacterial taxa, microbial KEGG enzymes predicted by PICRUSt2, rumen fermentation parameters, serum parameters and milk production phenotypes (Figures 7A,B and Supplementary Tables S10, S11). Based on the network’s betweenness centrality and closeness centrality, *Roseburia* and Erysipelotrichaceae_UCG-002 were identified as core bacterial taxa. Moreover, the relative abundance of three KEGG enzymes associated with carbohydrate metabolism: EC 3.2.1.135, EC 4.2.1.42, and EC 3.2.1.10 were positively correlated with rumen Pr concentrations (*p* < 0.05), which were positively linked to five milk production phenotypes (*p* < 0.05). In addition, the positive interaction network of bacteria associated with milk production was positively correlated with two lipid metabolism-related enzymes, EC 3.1.2.21 and EC 3.1.2.20 (*p* < 0.05). These two enzymes were also positively correlated with five milk production phenotypes (*p* < 0.05).

Using a similar correlation-based network for MY-associated rectum bacteria, functional enzymes, fermentation parameters, serum parameters and milk production phenotypes (Supplementary Tables S12, 13 and Supplementary Figure S6), positive correlations were observed between the relative abundances of *Moryella* and the Christensenellaceae_R-7_group within the Firmicutes (*p* < 0.05). *Moryella* abundance was positively correlated with EC1.1.1.303 (*p* < 0.05), while Christensenellaceae_R-7_group abundance was positively correlated with EC1.1.1.4 (*p* < 0.05). Both EC1.1.1.303 and EC1.1.1.4 were negatively associated with rectum Bu concentrations, which were negatively correlated with serum GLB concentrations (*p* < 0.05). Notably, serum GLB concentrations were inversely associated with five milk production phenotypes (*p* < 0.05).

### 3.7 Classification of milk production in long-lived cows using rumen and rectum microbiota and phenotypic data in random-forest plots

**Figure 8:**
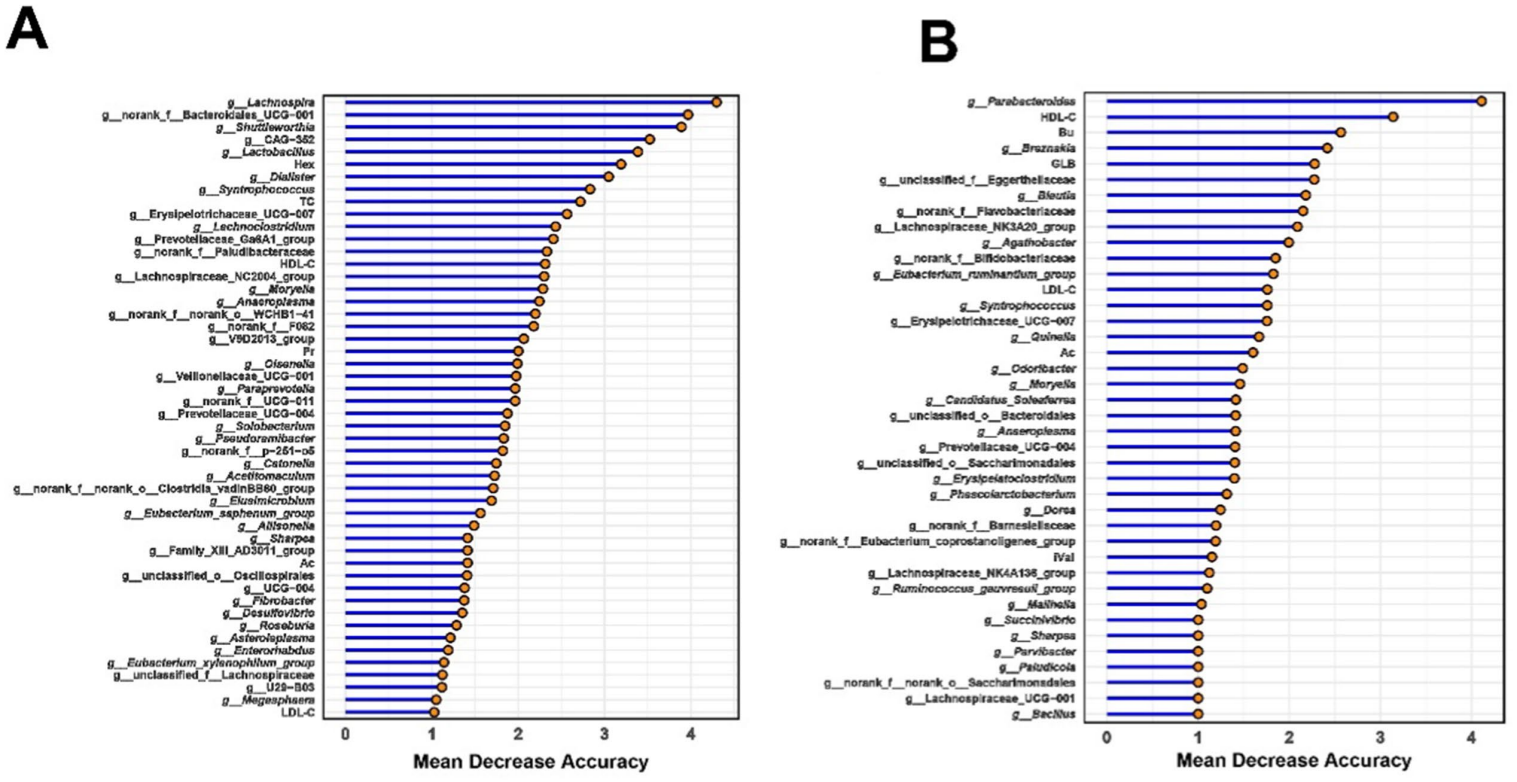
Random forest-based classification of milk yield in long-lived cows using **(A)** rumen and **(B)** rectum microbiota and phenotypic data. Important phenotypes were ranked by mean decrease in accuracy for classifying LH and LL groups. The classification model integrated SCFAs, serum parameters, and bacteria data to differentiate milk production categories using a random-forest algorithm.

Using rumen bacteria, rumen SCFAs, and serum parameters, a random forest model was applied to predict long-lived dairy cows with high production efficiency. The relative abundances of six bacterial taxa, *Lachnospira*, norank_f__Bacteroidales_RF16_group, *Shuttleworthia*, CAG-352, *Lactobacillus*, and *Dialister*, combined with rumem Hex concentration achieved the highest discrimination ability between LH and LL cows (AUC = 0.92, Figure 8A; Supplementary Figure S7A). Similarly, for rectum bacteria, rectum SCFAs, and serum parameters, the relative abundance of *Parabacteroides*, *Breznakia*, and unclassified_f__Eggerthellaceae, together with serum HDL-C, GLB, and rectum Bu concentrations, achieved near-perfect discriminatory power between LH and LL cows (AUC = 0.99, Figure 8B; Supplementary Figure S7B).

## 4 Discussion

The gastrointestinal microbiota play crucial roles in maintaining milk production and health of dairy cows (Xue et al., 2018; Brulin et al., 2024) by directly influencing MY, productive lifespan, and lifetime productivity (Pinedo et al., 2010). Current research on dairy cow lifetime productivity primarily focuses on integrating pedigree with phenotypic and genomic data (Clasen et al., 2017; Zhang H. et al., 2024), which is useful, but the role of the gastrointestinal microbiota needs to receive similar attention. In this study, we compared the rumen and rectum bacterial communities and host phenotypes of high-yield cows and low-yield cows with long-term productive lifespans. The data show that rumen bacteria can modulate carbohydrate and lipid metabolism, thereby influencing milk production (Figures 5–7). Rectum bacteria can potentially affect milk production phenotypes by influencing rectum butyrate production (Supplementary Figures S4–S6).

The adaptation of dairy cows to high-energy total mixed rations (TMR) during peak lactation increases the Firmicutes: Bacteroidetes ratio, enhancing energy utilization efficiency. Within the Firmicutes phylum, bacteria such as the Lachnospiraceae, which ferment high energy substrates to produce acetate—a key precursor for milk fat synthesis—showed increased abundance with a positive correlation to milk fat yield (Jami et al., 2014; Wang et al., 2021). *Prevotella*, a member of the phylum Bacteroidetes, known for its ability to ferment carbohydrates and proteins, also dominated the microbial community (Betancur-Murillo et al., 2022). This enhanced the adaptation of rumen bacteria in high-yielding cows to lactation diets results in a more specialized and simplified microbial composition, reducing bacterial richness (Ogata et al., 2019a; Rivera-Chacon et al., 2024). Our findings also confirmed that long-lived high-yielding cows exhibit lower rumen bacterial richness (Figure 1A), reflecting their superior adaptation to lactation-specific diets. Moreover, no significant difference was observed in the rectum microbiome α-diversity in this study (Figure 1B), which may be due to the fact that the rectum microbiome is likely more influenced by long-term adaptation and is generally more stable compared to the rumen microbiome (Ogata et al., 2019b; Wang et al., 2023a).

Inflammatory levels in dairy cows increase with age, with older cows exhibiting significantly elevated levels of inflammatory cytokines such as TGF-β, TNF-α, and IL-10 (Zhang et al., 2019). In addition, high-yielding dairy cows experience greater metabolic stress, leading to increased concentrations of AST and ALB in the serum (Amin et al., 2022). In this study, no significant differences were observed between LH and LL cows in serum energy metabolism indicators, oxidative stress biomarkers, or inflammatory and immunological markers, and all measurements remained within normal ranges. This may be attributed to the good health status of cows with long-term productive lifespans, as cow health is an important determinant of productive longevity (Pinedo et al., 2010). Moreover, LH cows had significantly higher levels of TC, HDL-C, and LDL-C compared to LL cows, while TG and GLB levels were significantly lower in LH cows. Most lipids in milk exist in the form of milk fat globules, with cholesterol being a crucial component of these globules and also influencing their size (Maheshwari et al., 2024). LH cows exhibited higher MFY, and the demand for cholesterol in mammary epithelial cells was also higher; however, only 20% of the cholesterol in milk is synthesized in the mammary gland (Long et al., 1980). The majority of cholesterol in the mammary gland originates from liver-produced TC transported via the bloodstream (Viturro et al., 2009). LDL is endocytosed by LDL receptors such as CD36 on mammary epithelial cells, facilitating the transport of cholesterol (LDL-C) into mammary cells (Bionaz and Loor, 2008). HDL can collect excess cholesterol from peripheral tissues and transport it back to the liver or transfer it to LDL (Rader and Hovingh, 2014). HDL-C levels partially reflect HDL cholesterol transport function and are inversely associated with the incidence of cardiovascular diseases (Nazir et al., 2020). The increase in TC, HDL-C, and LDL-C in the serum of LH cows reflects their heightened demand for cholesterol and mobilization of other lipids. Triglycerides carried by VLDL are the primary source of fatty acids for mammary epithelial cells. TGs are hydrolyzed by lipoprotein lipase (LPL) on the surface of mammary endothelial cells into fatty acids for mammary utilization, with LPL activity and expression significantly increasing during lactation (Fielding and Frayn, 1998; Bionaz and Loor, 2008). The observed decrease in TG levels in this study may result from elevated LPL activity in LH cows. Elevated GLB levels are often associated with chronic inflammation (Sadat et al., 2023; Stanojević et al., 2023), indicating an increased risk of inflammation in LL cows (Zhang et al., 2019).

LH cows also have higher Pr concentrations in the rumen, which is positively correlated with higher milk production phenotypes. Previous research showed that rumen Pr concentration was positively correlated with MY (Xue et al., 2018). Heritable rumen microbes can reduce the A/P ratio to increase ECM production (Zhang C. et al., 2024). Consistent with previous studies, rumen-enriched core bacteria associated with milk production phenotypes, such as *Syntrophococcus*, *Lachnospira*, *Shuttleworthia*, Erysipelotrichaceae_UCG-2, and *Roseburia*, demonstrated significant positive correlation in this study with rumen Pr concentrations (Figure 1G).

*Syntrophococcus* and *Lachnospira* are primarily Ac-producing bacteria, while Erysipelotrichaceae_UCG-2 produces both lactate and Ac. Moreover, some Ac-producing bacteria, such as *Syntrophococcus*, can also produce CO₂ (Rode et al., 1981; Doré and Bryant, 1990). The production of lactate and CO₂ provides substrates for Pr-producing bacteria to synthesize propionate (Gonzalez-Garcia et al., 2017). *Shuttleworthia* and *Roseburia* produce butyrate and CO₂ (Zhao Y. et al., 2023; Zhang X. et al., 2024), thereby helping to maintain microbial homeostasis in the rumen environment and supporting propionate production (Ungerfeld, 2020). Butyrate serves as an energy source for rumen epithelial cells, promoting growth and development, and facilitating the absorption of SCFAs (Aschenbach et al., 2019). Previous studies suggested that the symbiotic relationships among these bacteria synergistically optimize energy flow in the rumen, promoting its development in calves and improving production performance in adult dairy cows (Hao et al., 2021; Kong et al., 2022). A multi-tiered network analysis has demonstrated that these bacteria can enhance the decomposition of polysaccharides such as pullulan, maltose/dextrin, and dextran by influencing five carbohydrate-related enzymes enriched in high-lactation dairy cows. This supports glycolysis and Pr production, thereby improving productivity (Figure 7A). Interestingly, it has also been found that these bacteria can influence two enzymes related to lipid metabolism, supporting a milk-producing phenotype (Figure 7B). These enzymes facilitate the production of medium-chain fatty acids (MCFAs) while suppressing the utilization of medium- and long-chain fatty acids (M/LCFAs) in the rumen. The inhibition of M/LCFA utilization in the rumen not only allows for a greater escape of M/LCFAs into the small intestine but also preserves functional fatty acids for milk fat synthesis. An increased flow of M/LCFAs to the small intestine for utilization can reduce the risk of excessive fat mobilization in high-producing dairy cows (De Beni Arrigoni et al., 2016; Bionaz et al., 2020). In the later stages of a cow’s productive lifespan, lipid metabolism regulated by the rumen microbiome appears to play a crucial role in sustaining production performance. Targeted modulation of lipid metabolism through microbiome interventions may represent a novel strategy for maintaining the productivity of dairy cows with extended productive lifespans.

Bu concentrations were elevated in the rectum contents of LH dairy cows (Figure 3B), while the Christensenellaceae_R-7_group and *Moryella* exhibited reduced abundance (Supplementary Table S4). These bacterial groups were negatively correlated with Bu level, and positively correlated with serum GLB (Figure 3D). Christensenellaceae R-7 group is known as a butyrate producer and is enriched in the gut of beef cattle suffering from ruminal acidosis. In high-LH dairy cows, the decreased abundance of the Christensenellaceae R-7 group appears to indicate a healthier hindgut microbial environment (Wu et al., 2024). *Moryella* has been demonstrated to coexist with pathogenic bacteria and is enriched in the rumens of cows with mastitis (Yuan et al., 2021; Png et al., 2022; Guo et al., 2024). A reduction in the abundance of the Christensenellaceae R-7 group and *Moryella* supports the maintenance of a balanced rectum microbiome, thereby favoring the production of SCFAs by hindgut microbes—especially butyrate, which plays a crucial role in maintaining intestinal barrier function and exerting anti-inflammatory effects. Our multi-layered network analysis revealed positive correlations between the relatively less abundant genera of the Christensenellaceae_R-7_group and *Moryella*, and two less abundant enzymes involved in the (R,R)-butane-2,3-diol synthesis pathway. These enzymes work together to facilitate the conversion of pyruvate to (R,R)-butanediol (García-Quintáns et al., 2008; Radoš et al., 2015). The decreased abundance of the two bacterial groups and enzymes may indirectly promote Bu synthesis by reducing the conversion of propionate to (R,R)-butane-2,3-diol. Increased Bu levels in the rectum enhance the expression of tight junction proteins and aid in the repair of the mucosal epithelium, which reduces both intestinal and systemic inflammation (Peng et al., 2009; Liu et al., 2024), may result in lower serum GLB concentrations in LH cows (Supplementary Figure S6). Additionally, enhanced hindgut health benefits the absorption of S/MCFAs (Jørgensen et al., 1998). Machine learning methods have been widely used to distinguish ruminants with different production performances (Xue et al., 2022; Wang et al., 2023a). In this study, it was found that combining rumen and rectal bacteria with phenotypic indicators can accurately predict cows with sustained productivity. Notably, the random forest machine learning algorithm revealed that the concentration of Hex (a rumen fermentation indicator), together with five rumen bacteria—*Lachnospira*, norank_f__Bacteroidales_RF16_group, *Shuttleworthia*, CAG-352, Lactobacillus, and *Dialister*—can serve as key microbial markers for distinguishing between LH and LL cows, achieving an accuracy of 92%. Similarly, rectum *Parabacteroides*, *Breznakia*, and unclassified_f__Eggerthellaceae, together with serum HDL-C, GLB, and rectum Bu, can also serve as key microbial markers for distinguishing between LH and LL cows, with an accuracy as high as 99% (Figure 8; Supplementary Figure S7). However, further expansion of the sample size and the integration of other omics data such as genomics and metabolomics are necessary for this conclusion to be applied in practice.

**Figure 9:**
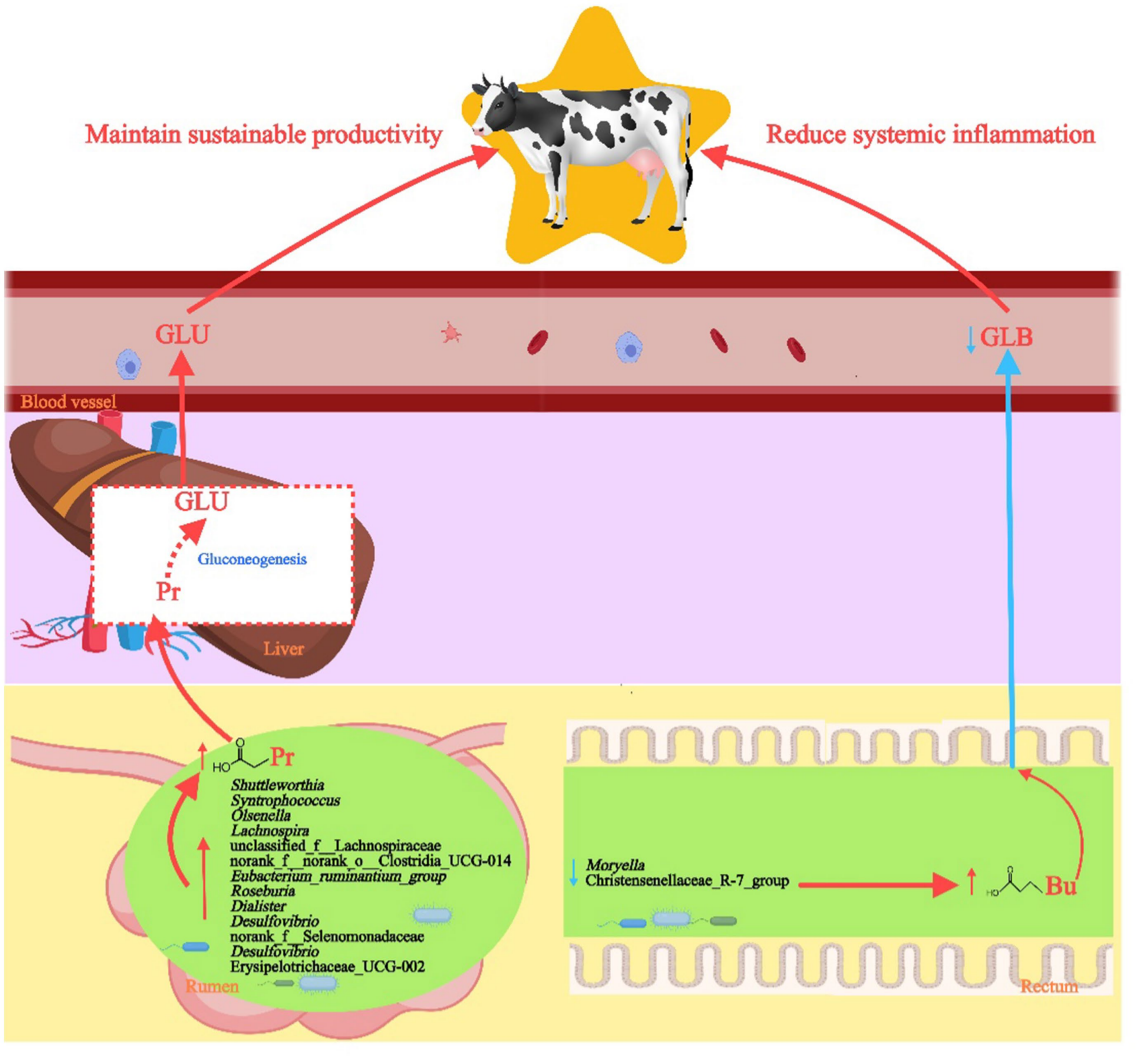
Diagram of the effects of rumen and rectum bacteria on the production performance of high-yielding and long-lived dairy cows. FFAs, free fatty acids; Gro, glycerol; M/LCFAs, medium and long-chain fatty acids; CM, chylomicron.

Although this study preliminarily investigated the effects of differential bacterial functions using PICRUSt2 predictions and correlation analysis, the inherent limitations of PICRUSt2 may result in discrepancies between predicted functions and actual biological processes (Sun et al., 2020). Therefore, future research should employ metagenomic sequencing to directly assess the presence and abundance of functional genes, providing comprehensive data for validation. Additionally, although all LH dairy cows in this study were managed under identical feeding and housing conditions to minimize non-genetic influences on the gut microbiome, host genetics play a crucial role in shaping and regulating the composition, function, and metabolites of the rumen microbiome through host-microbe interactions, which significantly affects lactation performance (Bai et al., 2024; Zhang C. et al., 2024). Future studies should explore how the host genotype modulates the composition of intestinal bacteria in cows and apply this knowledge to manipulating the gut microbiome by selective breeding of cows with long-term productive lifespans (see Figure 9).

## 5 Conclusion

In summary, this study found that rumen and rectum bacteria potentially influence the production performance of dairy cows with long-term productive lifespans through different pathways (Figure 9). Specifically, LH cows exhibit increased abundance of rumen bacteria with carbohydrate metabolism capabilities that promote Pr production, such as *Syntrophococcus*, *Lachnospira*, *Shuttleworthia*, Erysipelotrichaceae_UCG-2, and *Roseburia*, along with decreased abundance of key rectum genera in the Christensenellaceae_R-7_group and *Moryella*, which favors the production of Bu. The rumen and rectum microbiomes, along with serum phenotypes, can also serve as effective biomarkers for selecting high-yielding dairy cows with extended productive lifespans. These findings enhance our understanding of how different microbiomes affect the sustained productivity of dairy cows over their lifetimes and open avenues for future research aimed at breeding cows with long-term productive lifespans and extending the productive lifespan of the entire dairy cow population through microbiome selection and interventions.

## Data Availability

Raw sequencing data have been deposited in the National Center for Biotechnology Information (NCBI) under BioProject accession number PRJNA1246358.
