# Supplementary material for: Associations of rumen and rectum bacteria with the sustained productive performance of dairy cows
Source: Front Microbiol. 2025 Apr 29;16:1565034. doi: 10.3389/fmicb.2025.1565034 (PMC12069273; doi:10.3389/fmicb.2025.1565034)
Supplement: Supplementary file 1 [file Data_Sheet_1.docx]

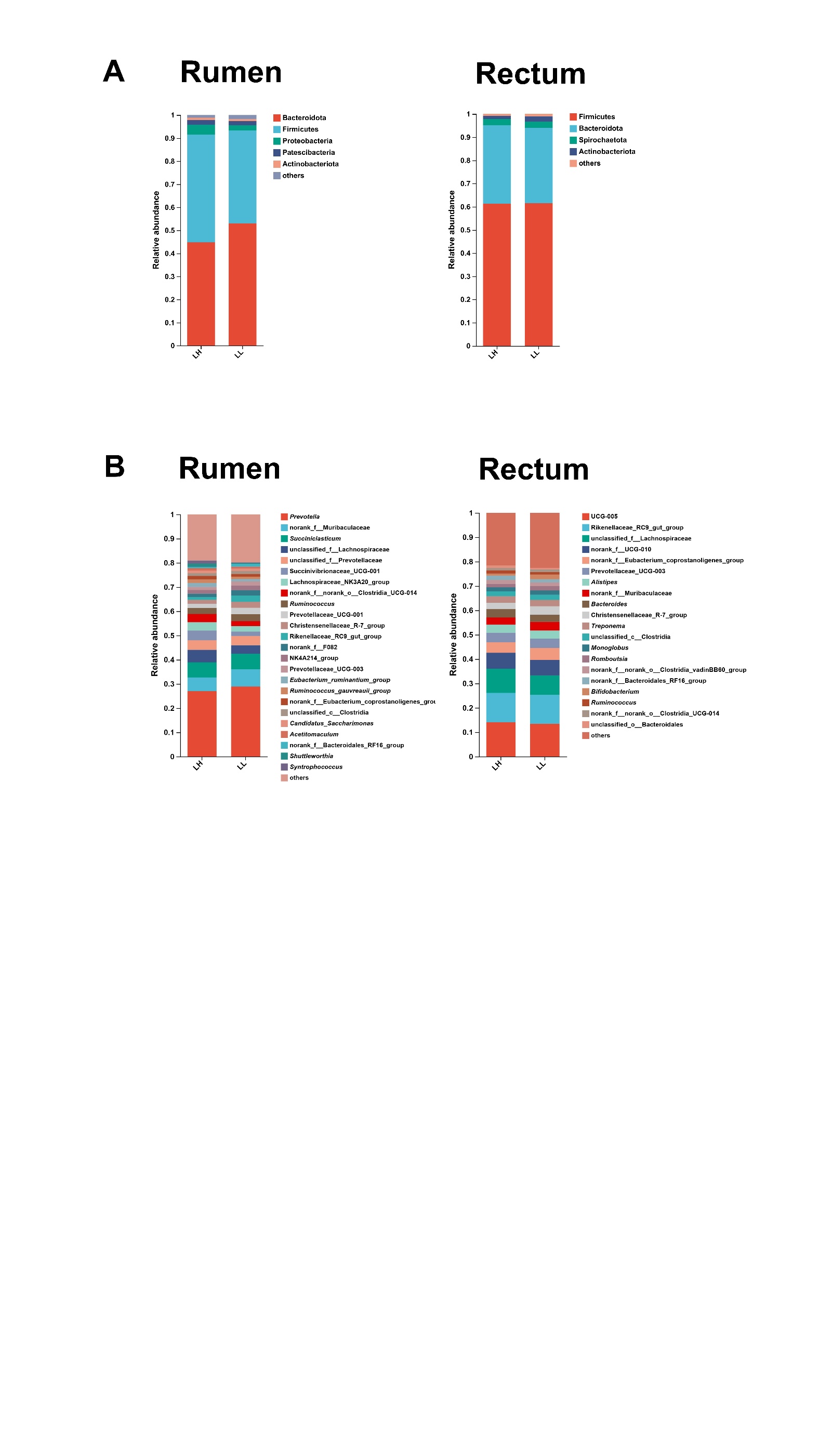


**Fig. S1 Bacterial composition in the rumen and rectum of LH and LL groups.** The relative abundances of the bacterial communities at the phylum (**A**) and genus (**B**) levels in the rumen and the rectum.


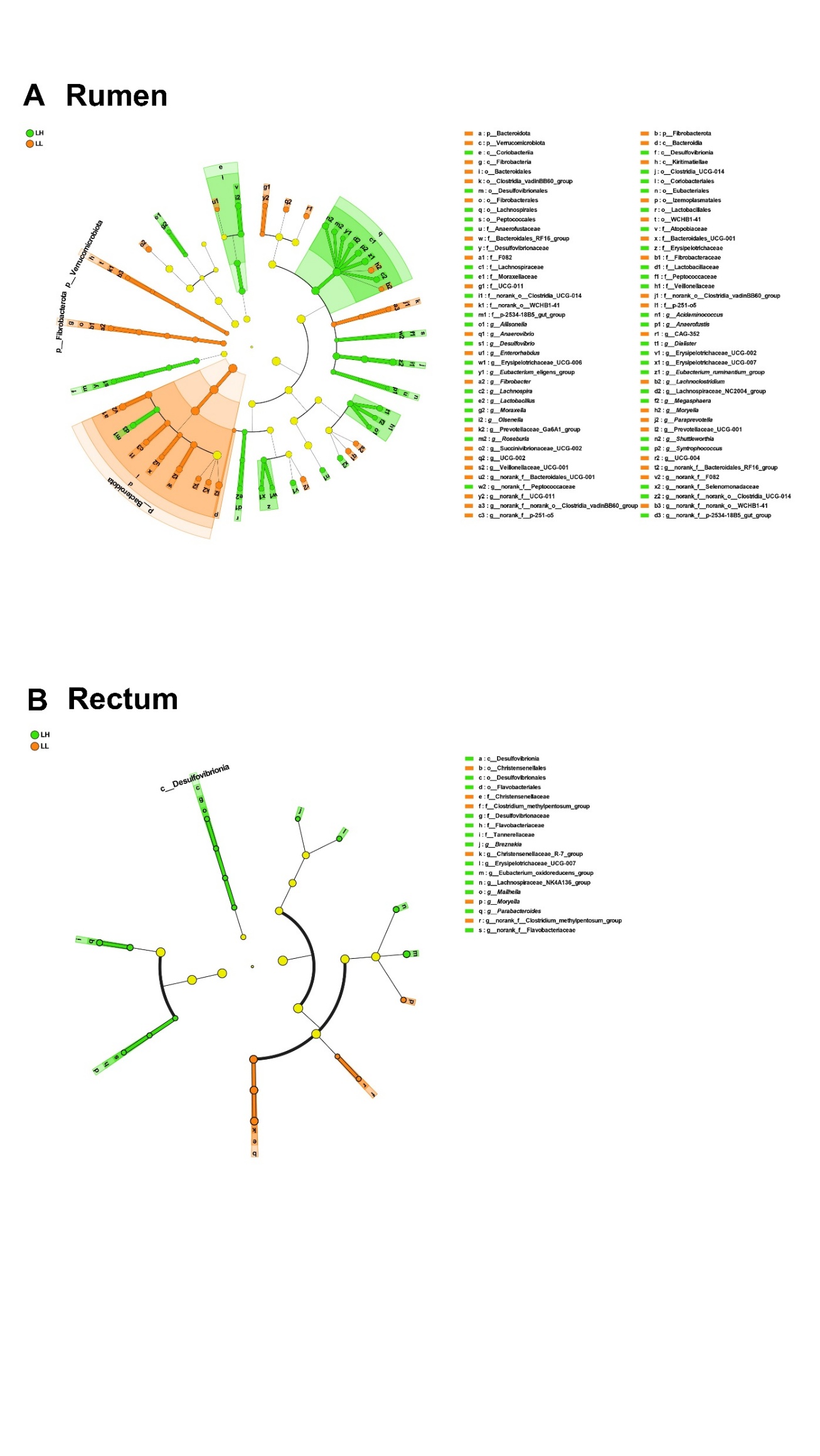


**Fig. S2 Differential bacterial taxa in the rumen and rectum between (A) LH and (A) LL groups.** Based on LEfSe analysis, the bacterial taxa with significant differential between the LH and LL groups were identified, and a taxonomic hierarchy tree was generated to display multiple classification levels. The significance threshold was set at an LDA > 2 and *P* < 0.05.


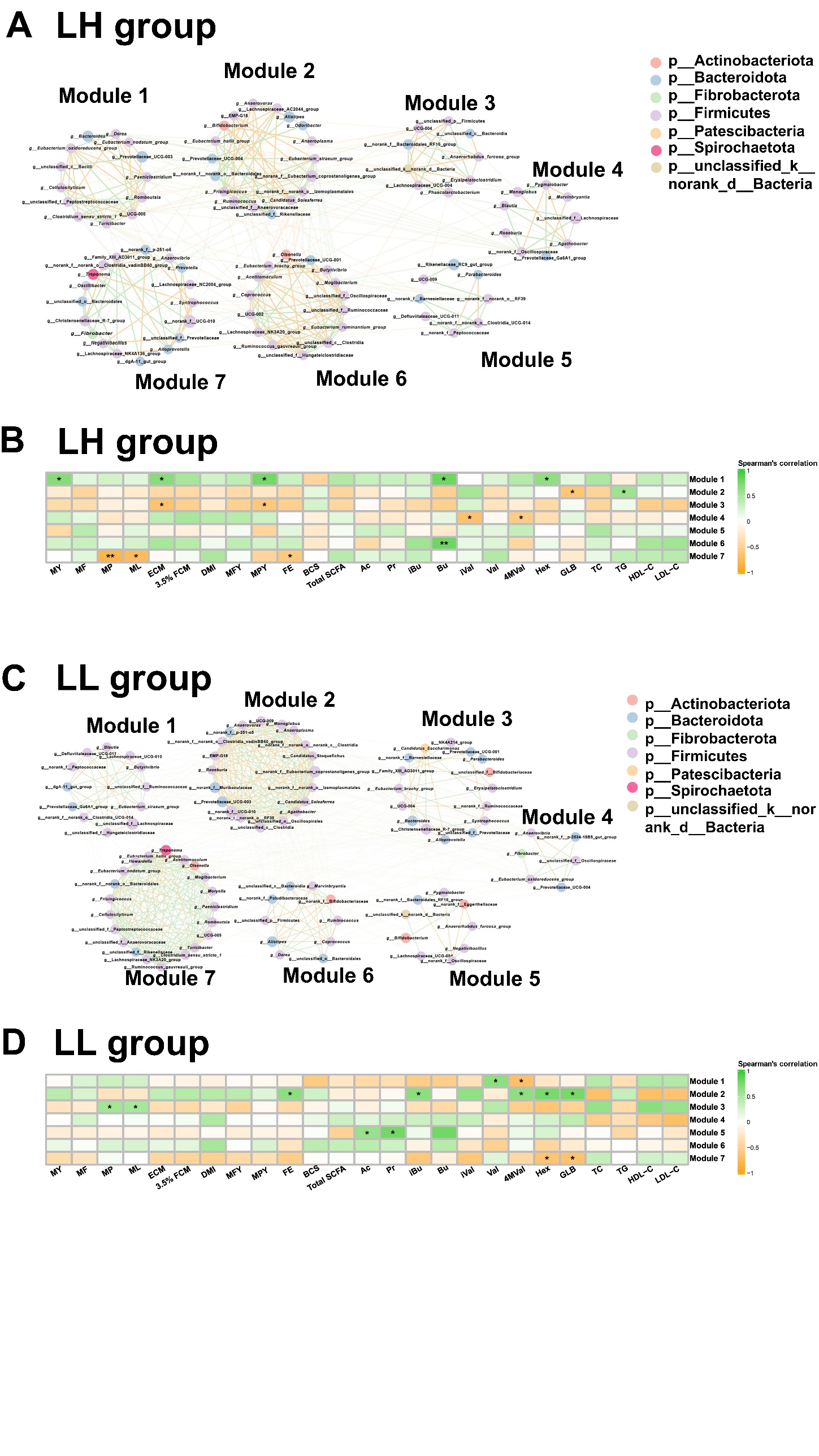


**Fig. S3 Co-occurrence networks and correlation analysis of rectum bacteria in LH and LL cows.** (**A** and **C**) Co-occurrence network of rectum bacteria at the genus level, with bacterial genera assigned based on their network roles in LH and LL cows (n = 10 per group). Nodes represent bacterial genera, with node size indicating the relative abundance of each genus. The color of the edges between nodes indicates positive (green) or negative (yellow) correlations (Spearman's |*r*| > 0.60 and *P* < 0.05). The thickness of the edges represents the magnitude of Spearman's |*r*|. (**B** and **D**) Correlation analysis on the rectum microbiota modules and phenotypes in LH and LL group. The color gradient represents the values of correlation coefficients (spearman’s correlation). *P* < 0.05*, *P* < 0.01**.


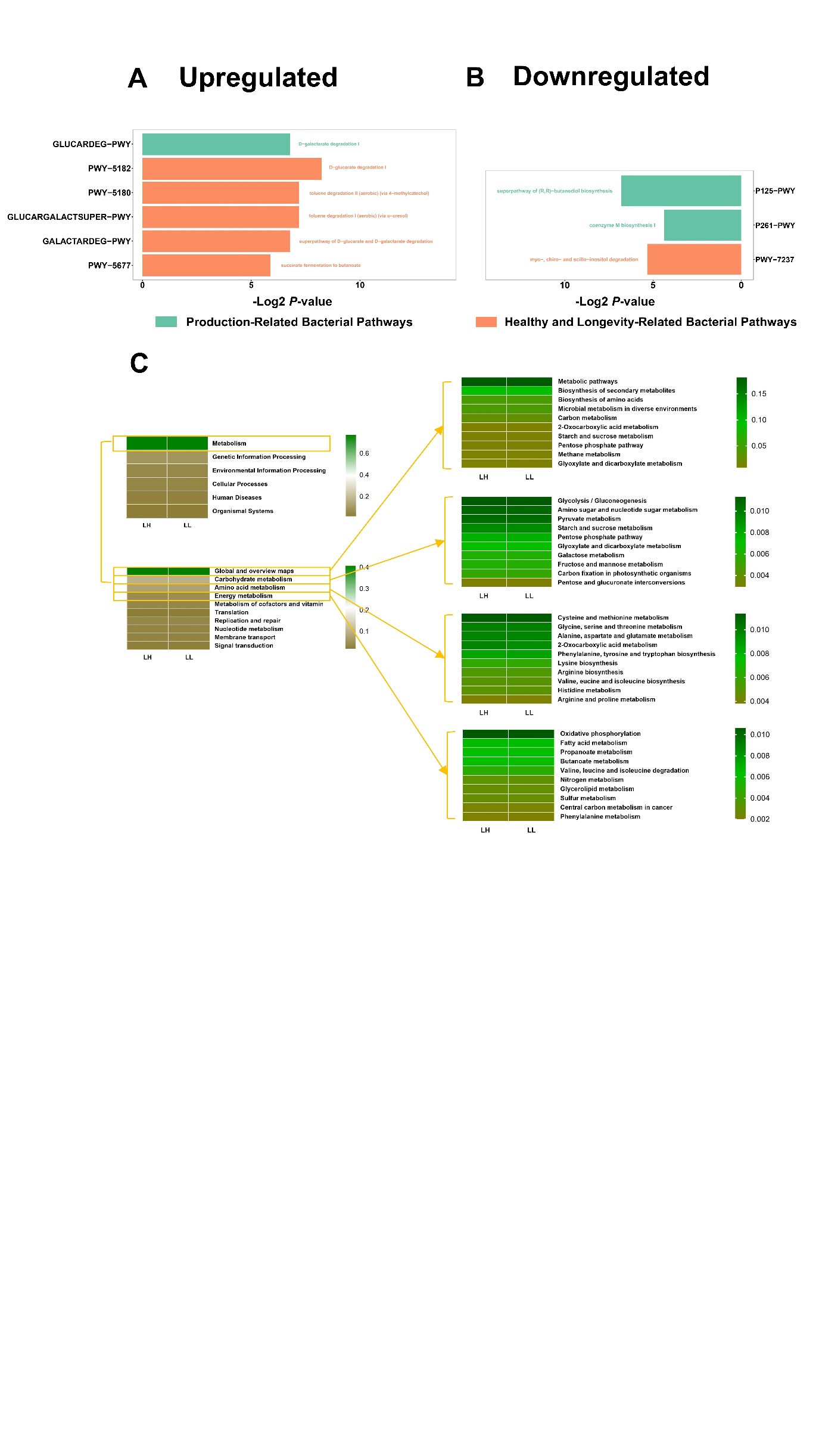


**Fig. S4 Functional predictions of rectum bacteria between LH and LL groups by PICRUSt2.** Relative abundances of rectum bacteria functional KEGG pathways level 1, level 2 and level 3 (top 10 of global and overview map, carbohydrate metabolism, amino acid metabolism, and energy metabolism). KEGG pathways were compared using Student’s *t*-tests.


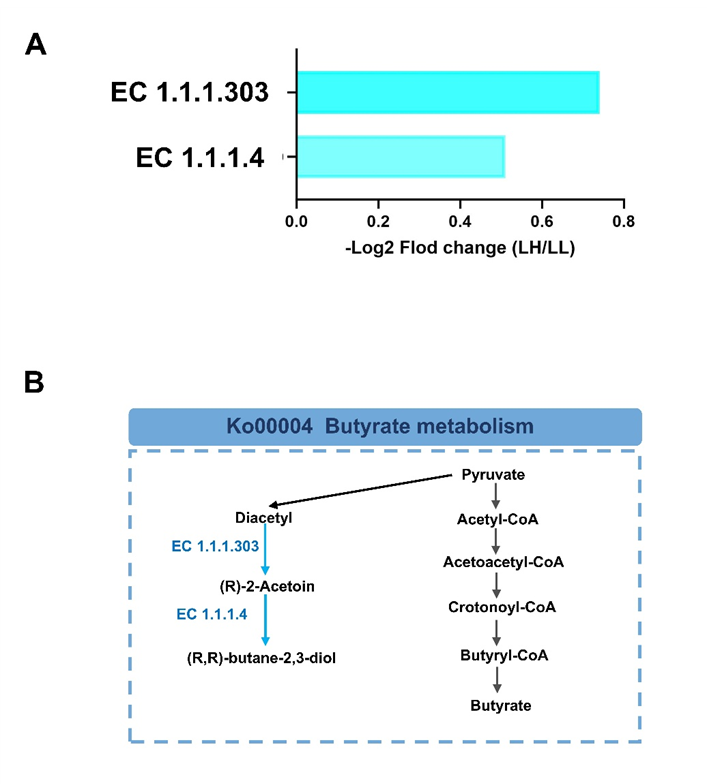


**Fig. S5 Differential rectum bacteria KEGG enzymes and metabolic pathways between LH and LL groups.** (**A**) Significantly different rectum KEGG enzymes between LH and LL groups (LDA > 2, *P* < 0.05). (**B**) Differential rectum microbial metabolic pathways between LH and LL groups. Blue names and arrows indicate KEGG Orthology and KEGG enzymes depleted in LH calves.


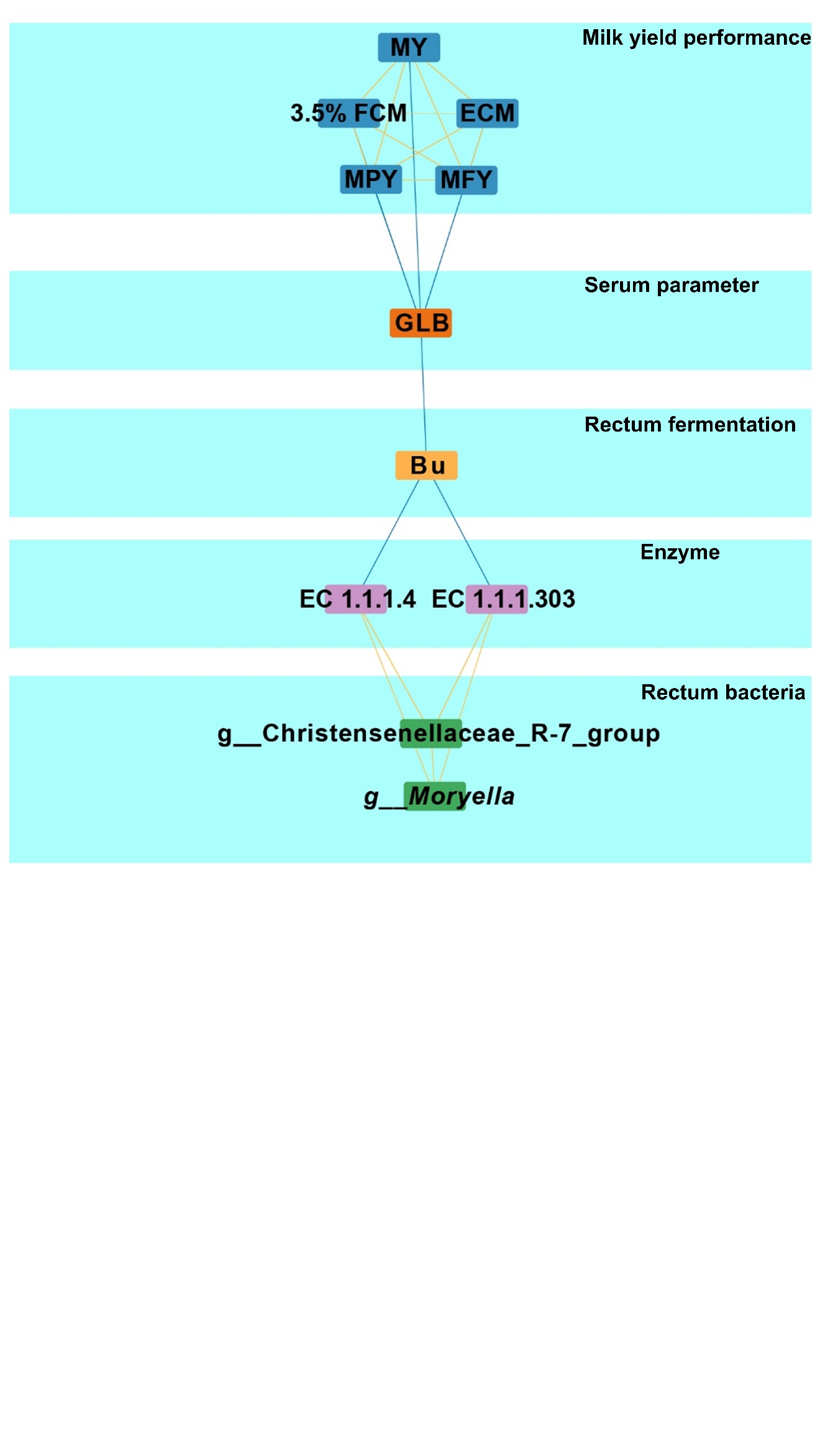


**Fig. S6 Multiplex networks revealed the relationships between rectum bacteria, rectum KEGG enzymes, rectum fermentation, serum parameter and milk production phenotypes.** Lines between two nodes represent the correlation, with a yellow line indicating a positive correlation and a blue line indicating a negative correlation (Spearman's |*r*|> 0.50 and *P* < 0.05).


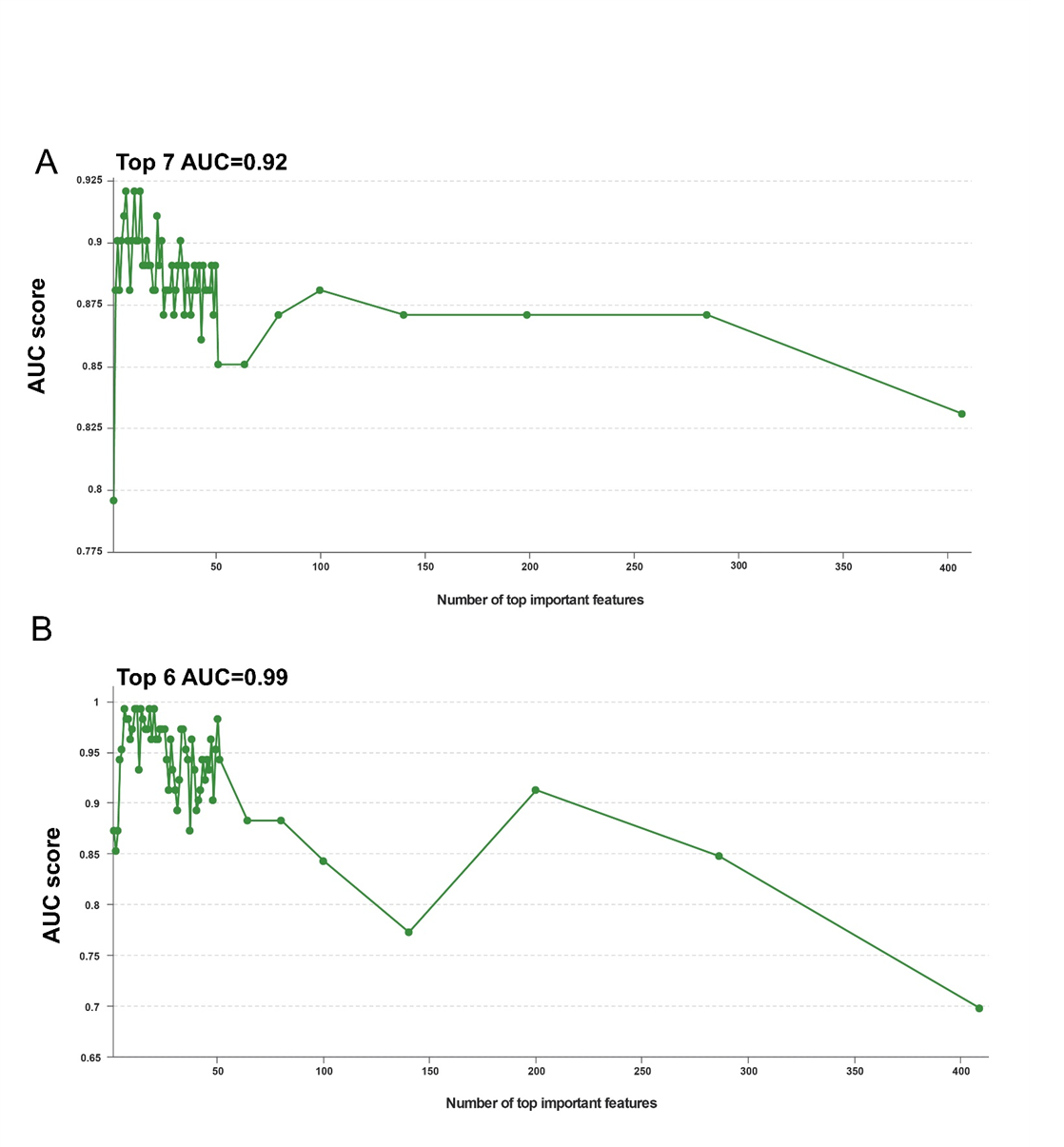


**Fig. S7 The predictive performance of the random forest model, using the AUC validation method, is evaluated based on different numbers of (A) rumen or (B) rectum bacteria genera and clinical factors.** The X-axis represents the top N variables selected by importance ranking, while the Y-axis indicates the AUC value of the model built with the corresponding variable set.
